# A reminder before extinction strengthens episodic memory via reconsolidation but fails to disrupt generalized threat responses

**DOI:** 10.1038/s41598-017-10682-7

**Published:** 2017-09-07

**Authors:** Marijn C. W. Kroes, Joseph E. Dunsmoor, Qi Lin, Michael Evans, Elizabeth A. Phelps

**Affiliations:** 1 Department of Psychology, New York University, New York, NY 10003 United States; 2 Center for Neural Science, New York University, New York, NY 10003 United States; 3 Nathan Kline Institute, Orangeburg, NY 10962 United States; 4 University of Texas at Austin, Department of Psychiatry, Austin, TX 78712 USA

## Abstract

A reminder can temporarily renew flexibility of consolidated memories, referred to as reconsolidation. Pavlovian threat-conditioning studies suggest that a reminder can renew flexibility of threat responses but that episodic memories remain stable. In contrast, outside the threat-conditioning domain, studies testing memory for word lists or stories find that a reminder can renew flexibility of episodic memory. This discrepancy in findings leaves it unclear if episodic memories reconsolidate, or only Pavlovian responses. Here we unite the different approaches in the field and show that a reminder can retroactively strengthen episodic memory for Pavlovian threat-conditioned events, but that, in contrast to threat-conditioning studies with simple sensory stimuli, extinction after a reminder fails to prevent recovery of generalized threat responses. Our results indicate the episodic memories also reconsolidate, allowing strengthening of relevant memories. These findings also suggest that generalized threat responses and episodic memories are less susceptible to be modified by reminder-interventions procedures.

## Introduction

The traditional view of memory is that new memories are initially flexible and sensitive to interference but stabilize over time during a consolidation period after which they remain essentially unchanged^1^. Emerging research over the past decade or so has challenged this traditional view of memory by suggesting that an isolated reminder can momentarily renew the flexibility of a consolidated memory so that it requires stabilization processes to be maintained, referred to as reconsolidation for reviews see^2–6^. Another major accomplishment of memory research is the appreciation that there are multiple memory systems that underlie different forms of behavioural expression^7–10^. Although reconsolidation is posited as a general theory of memory^2, 3, 11^ it is unclear whether reconsolidation occurs in all memory systems.

The majority of evidence for reconsolidation comes from studies with laboratory animals that use Pavlovian threat (fear) -conditioning paradigms with simple sensory stimuli (e.g. a tone predicts electrical shock). These studies show that interventions following an isolated reminder can modify threat-related defensive responses, such as freezing, and reverse neural signatures of memory consolidation within the amygdala (e.g. local field potentials, immediate early gene expression, and AMPA receptor trafficking), a region critical for this behavioural expression of threat memory for reviews see^2, 3^. Mirroring these findings, research with humans using threat-conditioning paradigms with simple sensory stimuli (i.e. a picture predicts electrical shock) also show that interventions in combination with an isolated reminder can diminish threat-related defensive responses such as startle or sweat responses^12, 13^. Despite a diminishment of threat responses, participants in these studies can still report the contingency between the pictures and shocks^12, 14^, a form of memory expression that is thought to depend on the hippocampus^15^. This has led some researchers to suggest that interventions targeting reconsolidation are optimal therapeutic techniques because they only affect threat responses and leave episodic memory intact. However, in threat-conditioning studies with simple stimuli the demand on episodic memory is small, and conditional threat responses can be acquired in the absence of episodic memory^16–18^. What is more, measures such as defensive responses or explicit knowledge of stimuli-shock contingencies are limited assays. They provide a single measure of behavioral expression that reflects an aggregate over the entire history of learning experiences. They do not allow testing of episodic memory for the individual events of an experience. Thus it is unclear from threat-conditioning studies if an isolated reminder only renews flexibility of learned threat responses or also of episodic memories.

Outside of the threat-conditioning domain, studies in humans testing memory for word lists or stories reveal that interventions following a reminder can result in time-dependent modification of episodic memories^19–24^. The few studies that have investigated reconsolidation of such hippocampus-dependent memories yield more mixed results than threat-conditioning studies with studies showing weakening^20, 21, 24, 25^, strengthening^19–24, 26^, or updating^19–24^ of episodic memory following a reminder. Studies with laboratory animals have demonstrated reconsolidation of hippocampus-dependent spatial memories, using experimental paradigms such as contextual threat conditioning^27, 28^, inhibitory place avoidance^29^, conditioned place preference^30^, and the Morris water maze^31^. However these studies have not tested for the episodic details of the learning experience. Therefore, behavioural and neural evidence for reconsolidation in animals provide limited support to studies on reconsolidation of episodic memory in humans. Thus studies outside the threat-conditioning domain suggest that episodic memories can become flexible, but because of the difference in experimental paradigms it is unclear if findings on the flexibility of episodic memory in humans reflect the same reconsolidation mechanisms as revealed by studies with laboratory animals. Interestingly, to date no human reconsolidation study has comprehensively tested episodic memory for a Pavlovian threat learning experience.

The objective of the present study was to unite the reconsolidation literature and bridge the translational gap between animal and human studies by investigating whether a reminder can renew flexibility of episodic memory for threatening events. To achieve this, we probed the effect of an isolated reminder on episodic memory within a Pavlovian category threat-conditioning procedure^32^. During category threat-conditioning participants see unique items from a specific category that predict the possibility of an aversive outcome. As no single item repeats, only an items’ membership of a specific category has predictive value and threat responses generalize to novel items of the reinforced category. The use of novel unique items increases episodic memory demands and allows subsequent testing of episodic memory for the individual items^32, 33^. We integrated the category threat-conditioning procedure within a reminder-extinction paradigm^34^ that has provided supporting evidence that an isolated reminder can renew flexibility of threat memories in rodents and humans^13, 34–40^, but see refs 41–43.

**Figure 1:**
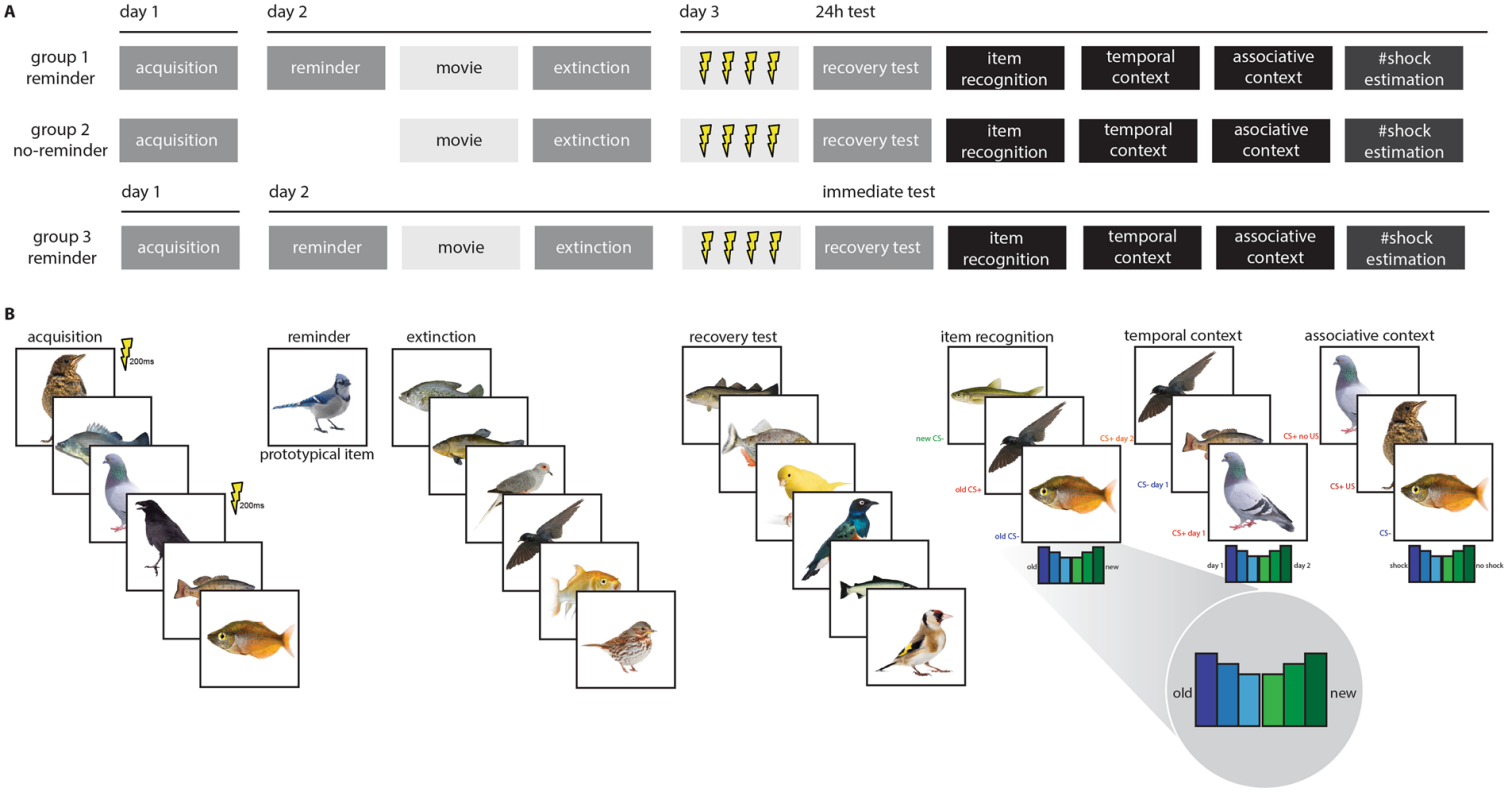
Design experiment. (**A**) Time line of experiment and (**B**) task depiction. Over 3 consecutive days participants were differentially conditioned to trial-unique items from a specific category (fish versus birds, counter-balanced across participants) signalling threat (CS+) of transcutaneous electrical shock (US) and unique items signalling safety (CS−) on day 1. On day 2, an isolated reminder was presented to one group (reminder group) but not the other (no-reminder group) by presentation of a novel trial-unique item that was the most prototypical exemplar of the CS+ category. Next, both groups underwent extinction to novel trial-unique items. On day 3, following reinstatement, the recovery of threat-related responses (skin conductance responses, SCR) to novel unique items was assessed. Critically, we tested participants’ recognition memory for the unique items, the items’ temporal context and associative context, and participants’ ability to estimate the number of received shocks. Responses during the memory tests were made on a six point likert scale (inset). In a follow-up control experiment, a group of participants followed the same procedure as the reminder group except that all memory tests were conducted after the reminder-extinction procedure on day 2. Images were obtained from Lifeonwhite.com. This figure is not covered by the CC BY license. © Lifeonwhite.com. All right reserved, used with permission.

In a between-subjects experiment over three consecutive days, forty-eight healthy adult participants were differentially conditioned to trial-unique items from a specific category signalling threat (CS+) of transcutaneous electrical shock (US) or safety (CS−) during acquisition on day 1 (see Methods and Fig. 1). The CSs were images of fish and birds and the category that served as CS+ and CS− was counterbalanced across subjects. On day 2, participants in one group (reminder group) but not the other group (no-reminder group) were presented with an isolated reminder of the CS+ category. After a 10-minute break both groups underwent extinction. Importantly, the reminder and extinction items were trial-unique and different from acquisition. On day 3, we tested if participants recognized which items (from day 1 and day 2) had been presented during the study (item recognition test). As episodic memory is defined by the ability to retrieve contextual information pertaining to a specific experience that occurred^9^, we also assessed the effect of a reminder on participants’ ability to remember when events had happened (temporal context test) and whether or not it had co-terminated with a shock (associative context test), and their ability to estimate how many shocks they had received (shock estimation test). Critically, this experimental design allowed us to test the effects of a reminder on items only ever encountered 24 h earlier and thereby isolate the retroactive effect of reconsolidation, the focus of our interest here. Besides tests of episodic memory we also probed the recovery of generalized threat responses following the reminder-extinction procedure.

The prediction from the threat conditioning reconsolidation literature was that a reminder would leave episodic memory unaffected. In contrast, the predictions from the episodic memory reconsolidation literature were that a reminder before new learning would weaken^20, 25^, strengthen^21, 26, 28^, or update^19^ episodic memory. Note that most studies on reconsolidation have used manipulations to impair normal brain function following a reminder to interfere with the behavioural expression of memory. However, reconsolidation itself is not dependent on post-reminder manipulations and in the absence of such manipulations reminders have been found to affect memory - particularly strengthening of hippocampal-dependent memories - via a reconsolidation process^26, 28, 44^. We found that an isolated reminder resulted in a retrograde and anterograde strengthening of episodic memory selective for item members of the reinforced category. But in contrast to threat-conditioning studies with simple sensory stimuli, we found that the reminder-extinction procedure did not prevent recovery of generalized threat responses. Finally, we conducted a follow-up control experiment in which a group of participants followed the exact same procedure as our reminder group, but here memory was tested immediately after the reminder-extinction procedure on day 2. This control experiment confirmed that the strengthening of episodic memory by a reminder is time-dependent, consistent with a reconsolidation phenomenon^2^.

## Results

### An isolated reminder selectively strengthened recognition memory

**Figure 2:**
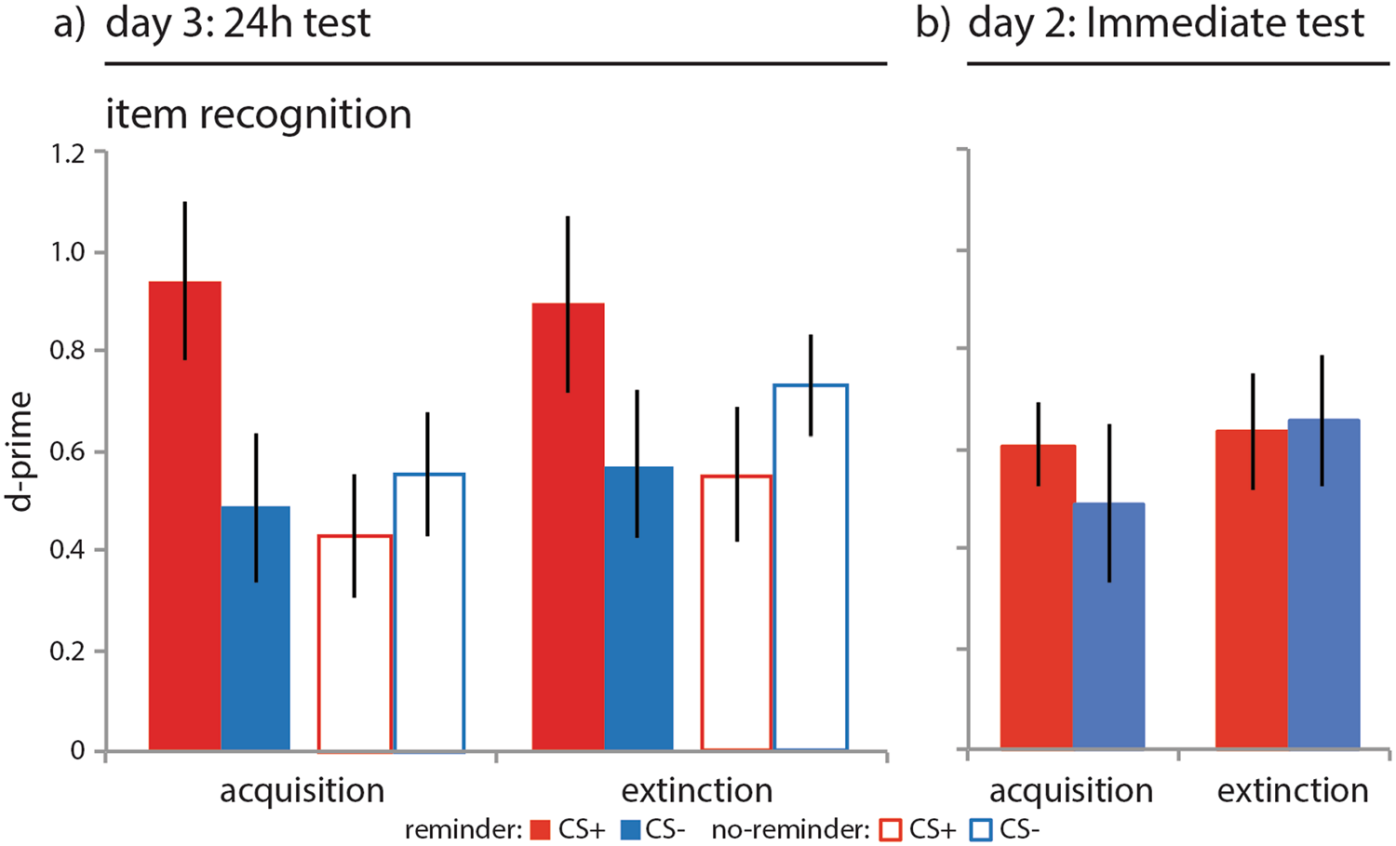
Results item recognition memory. An isolated reminder resulted in better item recognition specific to items from the CS+ category after 24 h but not immediately. (**a**) Reminder group (solid bars, N = 18), no-reminder group (open bars, N = 20), CS+ (red), CS− (blue), acquisition = items that were presented during acquisition, extinction = items that were presented during extinction, error bars reflect SEM. Across both groups participants were able to discriminate old from new items as revealed by one sample t-tests on d-prime scores (CS+: t(37) = 6.335, p < 0.001; 0.6711 ± 0.1059; CS−: t(37) = 5.483, p < 0.001; 0.5216 ± 0.0951). Follow-up independent samples t-test on difference scores ([CS+ acquisition + CS+ extinction] − [CS− acquisition + CS− extinction]) revealed greater differential item memory in the reminder group compared to the no-reminder group (t(36) = −3.050, p = 0.004; no-reminder: −0.2995 ± 0.2720; reminder: 0.7721 ± 0.2139). Follow-up paired samples T-test on the average d-prime scores across acquisition and extinction for CS+ and CS− items revealed better memory for CS+ items than CS− items in the reminder group (t(17) = 3.610, p = 0.002; CS+: 0.9158 ± 0.1207; CS−: 0.5298, ±0.1432) but no difference in the no-reminder group (t(19) = −1.101, p = 0.285; CS+: 0.4908 ± 0.1167; CS−: 0.6405 ± 0.0827). Planned t-test revealed better memory for CS+ than CS− items from acquisition (t(17) = 3.691, p = 0.002; CS+: 0.9386 ± 0.1570; CS−: 0.4868, ±0.1510) and extinction (t(17) = 2.571, p = 0.020; CS+: 0.8930 ± 0.1764; CS−: 0.5728, ± 0.1458) in the reminder but not the no-reminder group (p > 0.2). (**b**) For the reminder immediate memory test group (right panel, solid bars, N = 16) follow-up paired samples t-test on the average d-prime scores across acquisition and extinction for CS+ and CS− items revealed no difference in recognition memory (t(15) = 0.312, p = 0.759; CS+: 0.6855 ± 0.0611; CS−: 0.6458 ± 0.1314), and planned comparisons revealed no difference between CS+ and CS− items from acquisition and extinction (Ps > 0.6).

Our primary analyses of interest investigated the effect of an isolated reminder on subsequent episodic memory. During the item recognition test on day 3, participants again saw the trial-unique items from acquisition and extinction interspersed with an equal number of novel lure items. We assessed participants’ ability to recognize old items and discriminate them from new items by calculating a d-prime score for CS+ and CS− items from acquisition and extinction separately (Methods). A group (reminder, no-reminder) × task (acquisition, extinction) × CStype (CS+, CS−) repeated measures ANOVA on the d-prime scores revealed a CStype × group interaction (F_1, 36_ = 2.720, p = 0.004, η^2^ = 0.205) and no main effects or other interactions. Follow-up t-test indicated that the reminder group had better recognition memory for CS+ than CS− items from both acquisition and extinction whereas the no-reminder group showed no difference between the CS+ and CS− items (Fig. 2, also for additional statistics). To ensure that the group difference in recognition memory was an effect on discrimination ability and not confounded by differences in a response bias to endorse any item as ‘old’ we calculated a criterion score for the CS+ and CS− items from conditioning and extinction separately (Methods). We found no evidence for group differences on criterion scores (p > 0.2). The reminder group thus had better memory specifically for CS+ items from both acquisition and extinction and we found no evidence that this effect was confounded by differences in response bias.

### An isolated reminder selectively strengthened temporal context memory

**Figure 3:**
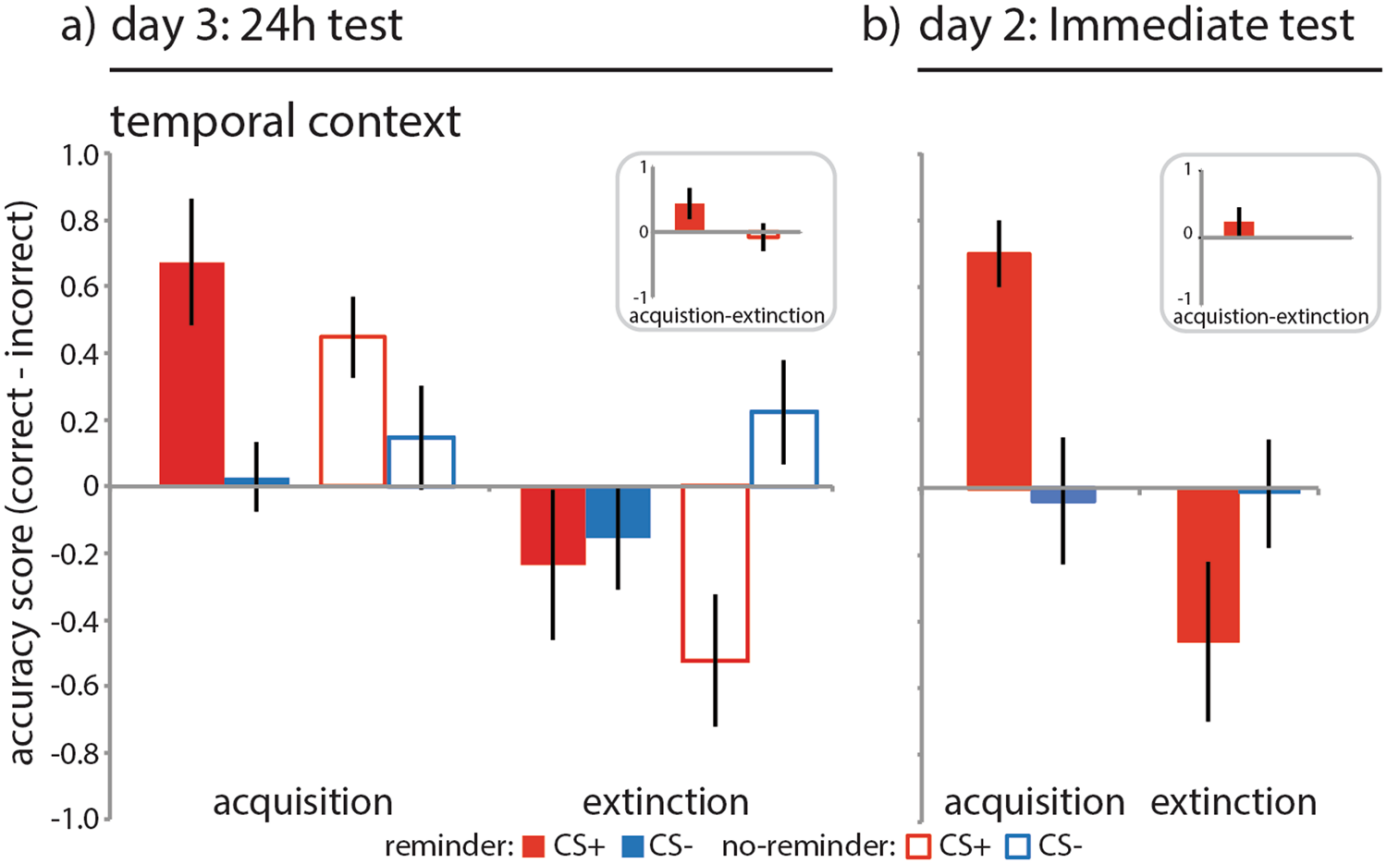
Results context memory. An isolated reminder resulted in better memory for the temporal context of items from the CS+ category. Reminder group (solid bars, N = 18), no-reminder group (open bars, N = 20), CS+ (red), CS− (blue), acquisition = items that were presented during acquisition, extinction = items that were presented during extinction, error bars reflect SEM. Follow-up one sample t-tests revealed that participants accurately indicated CS+ items from acquisition to be from day 1 (t(37) = 5.045, p < 0.001), but also inaccurately indicated CS+ items from extinction to be from day 1 (t(37) = −2.614, p = 0.013), and were not able to differentiate CS− items from acquisition (t(37) = 0.947, p = 0.350) and extinction (t(37) = 0.388, p = 0.701). Follow-up paired samples t-test revealed a difference in memory performance between CS+ items from acquisition and extinction (t(37) = 4.322, p < 0.001; CS+ acquisition: 0.5533 ± 0.1097; CS+ extinction: −0.388 ± 0.1485) but no difference for CS− items (t(37) = 0.290, p = 0.773; CS− acquisition: 0.0894 ± 0.0945; CS− extinction: 0.0441 ± 0.1137). Follow-up independent samples t-tests on temporal context memory scores averaged across CS+ items from acquisition and extinction (inset) revealed better memory in the reminder group than in the no-reminder group for the CS+ at trend (t(36) = −1.845, p = 0.073; no-reminder: −0.0395 ± 0.0778; reminder: 0.2128 ± 0.1193). (**b**) The reminder immediate test group (right panel, solid bars, N = 16) was able to accurately indicate the source of CS+ items from acquisition (t(15) = 7.114, p < 0.001: 0.7489 ± 0.1052) but not extinction nor did they inaccurately indicated items from extinction to be from acquisition (t(15) = −1.728, p < 0.105; reminder immediate group: 0. −0.4584 ± 0.2653) revealed by one-sample t-test. Averaging across CS+ trials from acquisition and extinction revealed that the reminder extinction group was overall not able to discriminate the source of items correctly (t(15) = 1.223, p = 0.240: 0.1452 ± 0.1188).

Having found an effect of an isolated reminder on item recognition we next assessed the effect of the reminder on contextual memory associated with the items. We measured participant’s ability to correctly indicate the temporal context of an item, i.e. whether an item had been presented on day 1 or day 2 by calculating a corrected memory score (correct - incorrect responses) for CS+ and CS− items from the acquisition and extinction separately (Methods). The corrected memory scores were submitted to a group (reminder, no-reminder) × task (acquisition, extinction) × CStype (CS+, CS−) repeated measures ANOVA. Memory performance for temporal context differed between groups (CStype × group: F_1, 36_ = 7.156, p = 0.011, η^2^ = 0.166). Follow-up t-tests revealed that the reminder group had better memory for the temporal context specifically for CS+ items (Fig. 3, also for additional statistics), although both groups were inclined to respond that CS+ items had been presented during acquisition irrespective of items’ actual temporal context and were unable to discriminate the temporal context of CS− items (task: F_1, 36_ = 14.185, p = 0.001, η^2^ = 0.283; task × CStype: F_1, 36_ = 10.129, p = 0.003, η^2^ = 0.220). The reminder group thus had better memory for the temporal context of CS+ items specifically.

### An isolated reminder selectively strengthened shock estimation memory

**Figure 4:**
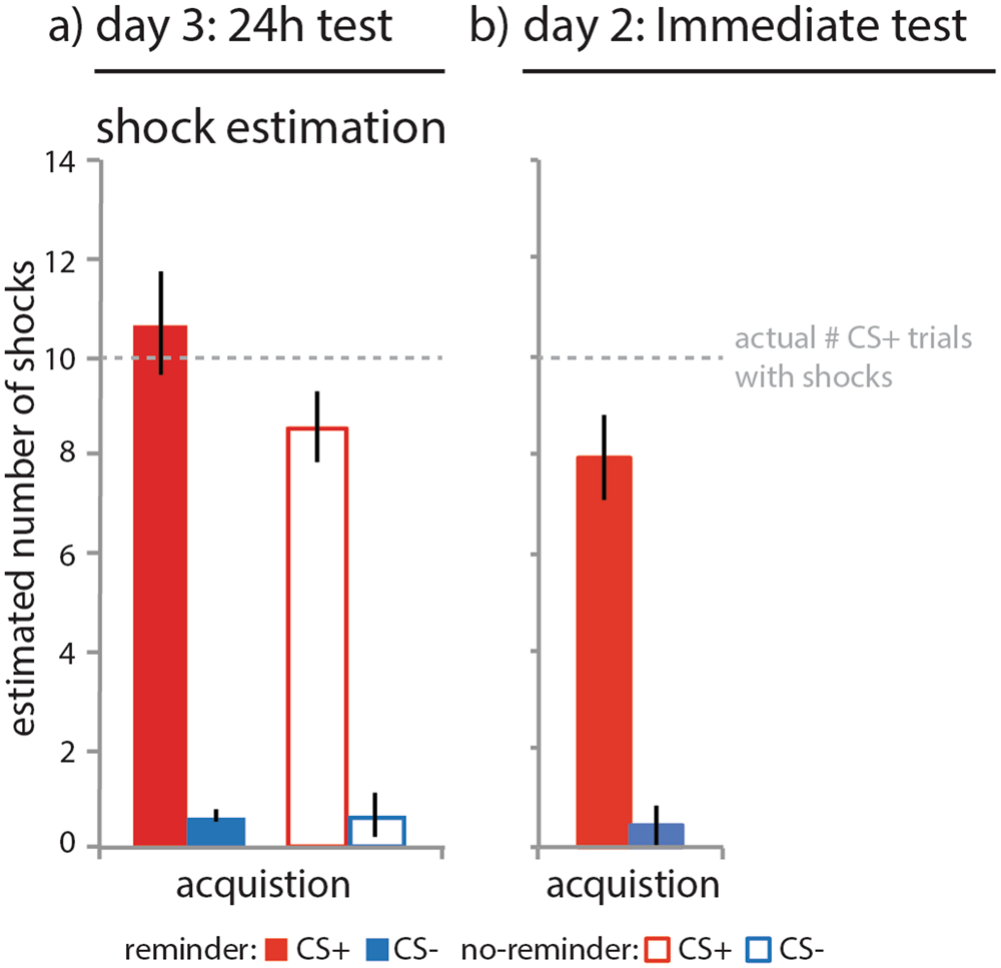
Results shock estimation memory. An isolated reminder affected the number shocks participants estimated (on day 3) to have followed CS+ exemplars on day 1 (i.e. during acquisition) in a time-dependent manner. (**a**) Reminder group (solid bars, N = 18), no-reminder group (open bars, N = 20), CS+ (red), CS− (blue), error bars reflect SEM. A follow-up paired samples t-test across groups revealed that on day 3 participants estimated having received more shocks following items of the CS+ category than CS− category during acquisition on day 1 (t(37) = 15.463, p < 0.001; CS+: 9.5526 ± 0.6341; CS−: 0.4211 ± 0.2314). A follow-up independent samples t-tests revealed that the reminder group estimated a higher number of shocks to have co-terminated with CS+ items compared to the no-reminder group at trend (t(36) = −1.709, p = 0.096; no-reminder: 8.550 ± 0.7089; reminder: 10.6667 ± 1.0416). Follow-up t-tests showed that the estimated number of shocks by the reminder group was not different from the actual number of shocks (t(17) = 0.640, p = 0.531; 10.6667 ± 0.1.0416). The no-reminder group, in contrast, marginally underestimated the number of shocks that had co-terminated with CS+ items (t(19) = −2.046, p = 0.055; 8.550 ± 0.7089). (**b**) The reminder immediate memory test group (right panel, solid bars, N = 16) also underestimated the number of shocks that had co-terminated with CS+ items as revealed by a one-sample t-test (*t*(15) = −2.117, p = 0.051; 8.0625 ± 0.91501).

We also asked participants at the end of the study to estimate the number of shocks that co-terminated with images of the CS+ and CS− category. Note that ten shocks co-terminated with the CS+ on day 1 only. The shock estimation scores were entered into a CStype (CS+, CS−) × group (reminder, no-reminder) repeated measures ANOVA. Participants in the reminder group estimated having received more shocks paired with the CS+ category than no-reminder group (CStype × group: F_1, 36_ = 5.408, p = 0.026, η^2^ = 0.111; Fig. 4, also for additional statistics). Follow-up t-tests revealed that the estimation of the number of shocks by the reminder group was not different from the actual number (*t*(17) = 0.640, p = 0.531; 10.6667 ± 0.1.0416). The no-reminder group, in contrast, marginally underestimated the number of shocks that were paired with CS+ items (*t*(19) = −2.046, p = 0.055; 8.550 ± 0.7089). Thus, the reminder group had better memory for the number of received shocks.

### An isolated reminder before extinction did not prevent the recovery of generalized threat responses

Having found that an isolated reminder strengthens episodic memory for threatening events, we also probed the effect of the isolated reminder before extinction on learned threat responses indexed by skin conductance responses. A group (reminder, no-reminder) × phase (early phase, late phase of task) × CStype (CS+, CS−) repeated measures ANOVAs on the mean SCRs revealed comparable acquisition of conditioned threat responses on day 1 (phase × CStype: F_1, 36_ = 23.431, p < 0.001, η^2^ = 0.394; see Methods, Supporting Tables 1 and 5 also for additional statistics). As we only included non-reinforced items in our analyses this indicated that threat responses generalized to novel items of the reinforced category, replicating previous research^32, 33^. On day 2 we observed retention and subsequent extinction of generalized threat responses in both groups (phase × CStype: F_1, 36_ = 3.356, p = 0.075, η^2^ = 0.085) that was complete by the last trial of extinction.

**Figure 5:**
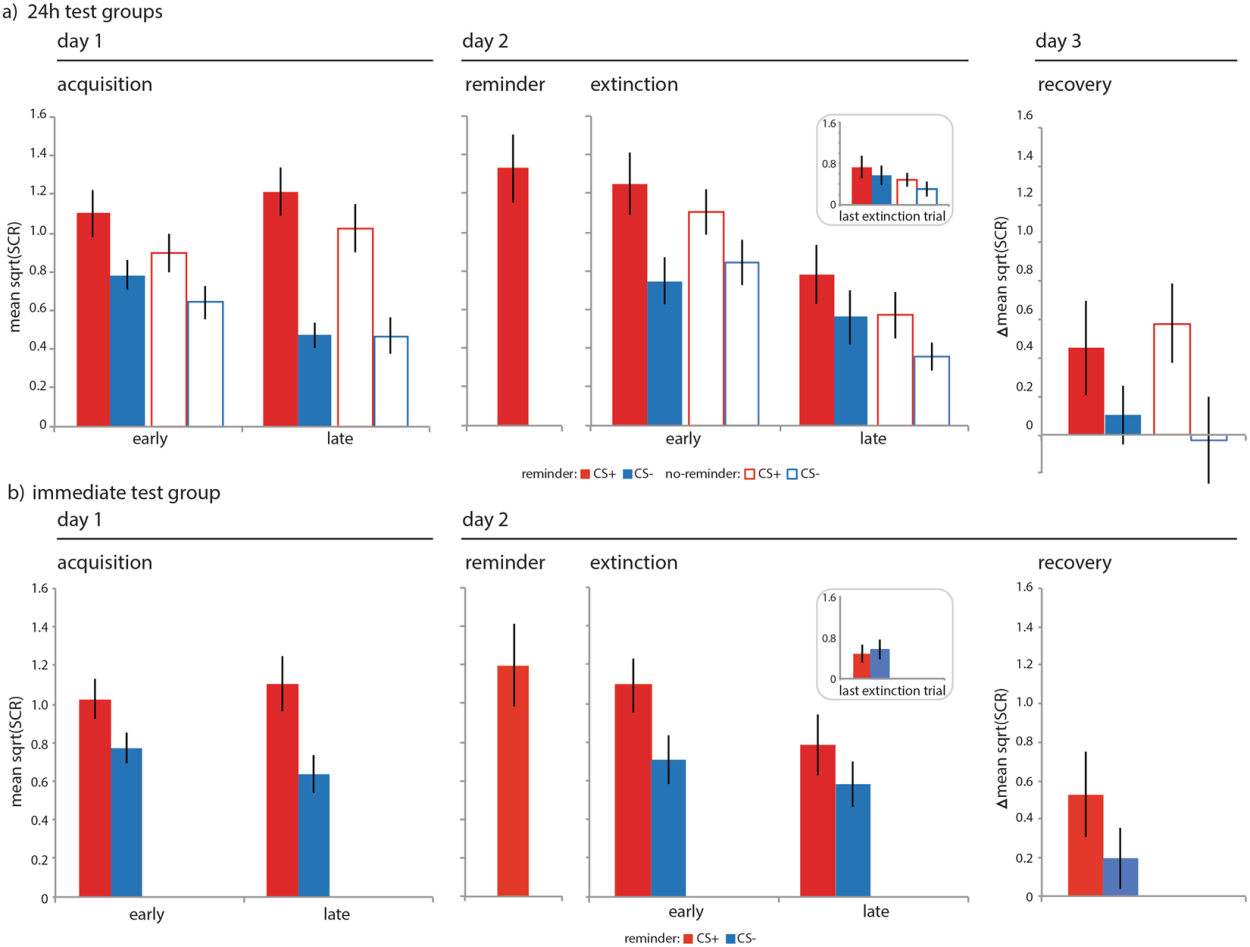
Results skin conductance responses. The aversive Pavlovian category threat-conditioning procedure resulted in the acquisition, retention and extinction of threat-related skin conductance responses. But an isolated reminder before extinction did not prevent the return of generalized threat responses one day later. (**a**) Reminder group (solid bars, N = 18), no-reminder group (open bars, N = 20), CS+ (red), CS− (blue), error bars reflect SEM. **Acquisition** (panel 1): Follow-up analyses revealed greater SCR to CS+ than CS− trial during the early phase (CS+ trial 1–5; CS− trial 1–10; t(37) = 4.806, p < 0.001; CS+: 0.994 ± 0.079; CS−: 0.710 ± 0.059) and late phase (CS+ trial 6–10; CS− trial 11–20; t(37) = 8.880, p < 0.001; CS+: 1.113 ± 0.088; CS−: 0.471 ± 0.057) across both groups, and the difference between SCR to CS+ and CS− trials was greater in the late than early phase (t(37) = −4.824, p < 0.001; early phase: 0.142, ± 0.030; late phase: 0.321 ± 0.036). **Reminder** (panel 2): The reminder trial evoked a SCR. **Extinction** (panel 3): Follow-up tests showed greater SCR to CS+ than CS− trials during the early phase (CS+ trial 1–6; CS− trial 2–6; t(37) = 5.949, p < 0.001; CS+: 1.1716 ± 0.0958; CS−: 0.7972 ± 0.0826) and late phase (CS+ trial 7–12; CS− trial 7–12; t(37) = 2.974, p = 0.005; CS+: 0.6716 ± 0.0965; CS−: 0.4534 ± 0.0784) across both groups, and the difference between SCR to CS+ and CS− trials became smaller in the late than early phase (t(37) = 1.779, p = 0.083; early phase: 0.3787 ± 0.0629; late phase: 0.2182 ± 0.0734). SCRs were no longer different between the last CS+ and CS− trial of extinction and no differences between groups were detected (CStype (CS+, CS−) × group (reminder, no-reminder) repeated measures ANOVA revealed no main effects or interactions: P > 0.1). **Recovery** (panel 4): A follow-up test revealed greater delta-recovery scores (change in responses for the last trial of extinction to the first trial of the recovery test) for the CS+ than CS− across both groups (t(37) = 3.422, p = 0.002; CS+: 0.5170 ± 0.1571; CS−: 0.0403 ± 0.1328). (**b**) The reminder immediate memory test group (lower panels, solid bars, N = 16) also showed acquisition, extinction, and recovery of generalized threat responses. **Acquisition** (panel 1): Follow-up analyses revealed greater SCR to CS+ than CS− trial during the early phase (CS+ trial 1–5; CS− trial 1–10; t(15) = 3.647, p = 0.002; CS+: 0.1.028 ± 0.106; CS−: 0.774 ± 0.080) and late phase (CS+ trial 6–10; CS− trial 11–20; t(15) = 4.680, p < 0.001; CS+: 1.105 ± 0.141; CS−: 0.638 ± 0.098) and the difference between SCR to CS+ and CS− trials was greater in the late than early phase (t(15) = −2.974, p = 0.009; early phase: 0.254, ± 0.070; late phase: 0.466 ± 0.100). **Reminder** (panel 2): The reminder trial evoked a SCR. **Extinction** (panel 3): Follow-up tests showed greater SCR to CS+ than CS− trials during the early phase (CS+ trial 1–6; CS− trial 2–6; t(14) = 3.201, p = 0.006; CS+: 1.0939 ± 0.1405; CS−: 0.7107 ± 0.1270) but not the late phase (CS+ trial 7–12; CS− trial 7–12; t(14) = 1.662, p = 0.119; CS+: 0.7855 ± 0.1575; CS−: 0.5805 ± 0.1160) and SCRs did not different between the last CS+ and CS− trial of extinction (inset; t(14) = −0.425, p = 0.667). **Recovery** (panel 4): A follow-up test revealed no differences in delta-recovery scores (change in responses for the last trial of extinction to the first trial of the recovery test) for the CS+ than CS− (t(14) = 1.197, p = 0.251; CS+: 0.5261 ± 0.2198; CS−: 0.1965 ± 0.1583). Yet, post-hoc one-sample t-test revealed recovery for the CS+ (t(14) = 2.393, p = 0.031) but not the CS− (t(14) = 1.241, p = 0.235).

Based on previous studies^13, 34, 37, 39^ that used threat-conditioning paradigms with simple sensory stimuli, we hypothesized that the reminder-extinction procedure might prevent the recovery of generalized threat responses. To test this hypothesis we assessed, following a reinstatement procedure where four unsignalled shocks were administered to increase arousal levels, the recovery of SCRs to novel trial-unique items of the CS+ and CS− category on day 3. We calculated a delta-recovery score as the increase in SCRs from the last trial of extinction to the first trial of the recovery test for the CS+ and CS− separately (Methods). We ran a CStype (ΔCS+, ΔCS−) × group (reminder, no-reminder) repeated measures ANOVA and found greater recovery of SCRs to the CS+ than CS− across both groups (CStype: F_1, 36_ = 11.377, p = 0.002, η^2^ = 0.240, with no other main effect or interaction (Fig. 5 for additional statistics) replicating a prior pilot experiment (see Supporting Information). Thus, in contrast to studies that used threat-conditioning paradigms with simple stimuli, an isolated reminder of memory for a threatening category before extinction did not prevent the recovery of generalized threat responses.

### The effects of an isolated reminder on memory are time-dependent

We conducted a follow-up control experiment identical to the design of the reminder group with the exception that all memory tests were conducted on day 2 immediately after the reminder-extinction procedure (see Fig. 1). We hypothesized that in the reminder group where we tested memory immediately we would not detect memory strengthening, as reconsolidation is a time-dependent process and would not yet have completed. A group (reminder, reminder immediate test) × task (acquisition, extinction) × CStype (CS+, CS−) repeated measures ANOVA on the d-prime recognition memory scores revealed a CStype × group interaction (F_1, 32_ = 4.410, p = 0.044, η^2^ = 0.121) and a main effect of CStype (F_1, 32_ = 6.663, p = 0.015, η^2^ = 0.171) and no other main effects or other interactions. Where we found that the reminder group at the 24 h memory test had better recognition memory for CS+ than CS− items from both acquisition and extinction (see above) the reminder immediate memory test group did not (Fig. 2b, also for additional statistics). For the temporal context memory scores a group (reminder, reminder immediate test) × task (acquisition, extinction) × CStype (CS+, CS−) repeated measures ANOVA revealed a main effect of task (F_1, 32_ = 14.558, p = 0.001, η^2^ = 0.313), CStype (F_1, 32_ = 4.427, p = 0.043, η^2^ = 0.122), and task × CStype interaction (F_1, 32_ = 10.379, p = 0.003, η^2^ = 0.245), but no differences between groups (Ps > 0.2). Yet where the reminder group at the 24 h test had been able to accurately indicate the source of CS+ items across acquisition and extinction, and did so better than the no-reminder group, the reminder immediate test group was not able to do so (Fig. 3b, also for additional statistics). For the shock estimation scores a CStype (CS+, CS−) × group (reminder, reminder immediate test) repeated measures ANOVA revealed a CStype × group interaction (F_1, 32_ = 4.441, p = 0.043, η^2^ = 0.122) and a main effect of CStype (F_1, 32_ = 152.346, p < 0.001, η^2^ = 0.826). Similar to the no-reminder group, the reminder immediate group underestimated the number of shocks that were paired with CS+ items at trend (Fig. 4b, also for additional statistics). For skin conductance response a group (reminder, reminder immediate test) × phase (early phase, late phase of task) × CStype (CS+, CS−) repeated measures ANOVAs revealed comparable acquisition of conditioned threat responses (phase × CStype: F_1, 32_ = 29.784, p < 0.001, η^2^ = 0.482), retention and extinction of threat responses (phase × CStype: F_1, 32_ = 8.220, p = 0.007, η^2^ = 0.210) that was complete by the last trial of extinction (Fig. 5b, also for additional statistics). For the delta-recovery score a CStype (ΔCS+, ΔCS−) × group (reminder, reminder immediate test) repeated measures ANOVA indicated greater recovery of SCRs to the CS+ than CS− across both groups (CStype: F_1, 32_ = 6.041, p = 0.020, η^2^ = 0.163, with no other main effect or interaction (Fig. 5 for additional statistics). Thus, the reminder strengthened episodic memory but did not prevent the recovery of threat responses in a time-dependent manner, consistent with reconsolidation phenomena.

## Discussion

Here we tested the effect of an isolated reminder on episodic memory for events associated with a Pavlovian threat learning experience. An isolated reminder of memory for a threatening category resulted in retrograde and anterograde strengthening of recognition memory that was selective to items related to the reinforced category. Testing for contextual memory associated with the items revealed that participants in the reminder group were better at estimating when items related to the reinforced category had been presented, although participants in both groups were inclined to indicate that items related to the reinforced category had been presented during acquisition regardless of whether the items had been presented during acquisition or extinction. A test of participants’ ability to estimate the number of shocks that had co-terminated with the category exemplars showed that the reminder group was better than the no-reminder group in estimating the number of shocks that had co-terminated with items of the reinforced category. In contrast to reminder-extinction studies with simple sensory stimuli, we found that a reminder of memory for a threatening category before extinction did not prevent the recovery of generalized threat responses. Thus an isolated reminder before extinction selectively and retroactively strengthened episodic memory for threatening events, but did not disrupt generalized threat responses as assessed by SCR. A follow-up control experiment confirmed that strengthening of episodic memory by a reminder was time-dependent, consistent with a reconsolidation explanation.

Our results suggest that a reminder can selectively strengthen episodic memory for threatening events. Most studies on reconsolidation have used manipulations to impair normal brain function (e.g. protein synthesis inhibitors or electroconvulsive shock) following a reminder to impair the behavioural expression of memory. As a result, reconsolidation is commonly considered a mechanism by which long-term memories are impaired or even ‘erased’. But both animal and human studies have shown that in the absence of interference a reminder can enhance episodic (-like) memory^20, 25, 26, 28, 45^. Here we extend those findings by showing that a reminder does not lead to a generic memory enhancement but affects relevant memories specifically. A reminder might, alternatively, allow updating or integration of an old memory with new information^19^. Although participants in our reminder group had better memory for items related to the reinforced category, we did not observe group differences in response bias. This is notable because the foils were similar to the target items, making the memory test difficult and likely requiring pattern separation processes see also ref. 46. Furthermore, this indicates that the reminder strengthened the memory representation of individual items and not simply increased the likelihood of retrieving any items of the threatening category as being old. The reminder group, moreover, was better at discriminating the temporal context of items of the reinforced category. Note, however that because the item memory test was conducted before the temporal and associative context test the former may have affected the latter even though we observed no correlations between critical performance measures on the different tests (P > 0.1). These results are most in line with an interpretation that a reminder resulted in selective strengthening of episodic memory (see Supporting Information for further discussion). Moreover, the isolated and unique reminder retroactively strengthened memory for items that were presented only once 24 hours earlier, and our control experiment confirmed that this effect was time-dependent. We therefore suggest that, akin to the proposed function of consolidation^47^, reconsolidation may allow for the competitive selection of relevant information to become permanently stored.

A critical concern is the mechanism that resulted in the strengthening of episodic memory. The design of our paradigm followed previous studies that have provided evidence for reconsolidation^13, 34, 37, 48^ and the retroactive strengthening of episodic memory we observed was dependent on a reminder and the passage of time, meeting critical criteria to demonstrate reconsolidation^21, 49, 50^ and mimic previous animal and human studies showing that a reminder can enhance episodic (-like) memory via reconsolidation processes^26, 28, 44^. We therefore suggest that the retroactive strengthening of episodic memory that we observed resulted from reconsolidation. Alternatively, the effect of a reminder on episodic memory may have resulted from a “testing effect”, reflecting re-encoding or strengthening of retrieval processes^51, 52^. First, it is important to note that the reminder did not explicitly test episodic memory. Further, our control experiment revealed that the reminder cue *enhanced* subsequent memory tested 24 h later compared to memory tested immediately after the reminder. In contrast, to the best of our knowledge, testing effects do not enhance memory at delayed tests relative to immediate memory tests but protect memory from forgetting^52^. In addition, in our study the reminder cue enhanced memory for non-tested items that were encoded 24 h earlier. We are unaware of reports showing that testing effects generalize to non-cued items unless they are integrated during study^53, 54^. Finally, the reminder group showed more accurate episodic memory and not simply an increased likelihood to retrieve any items of the threat category as old, suggesting that the reminder strengthened individual memory representations and not the retrieval likelihood of the category. Unexpectedly the reminder cue also resulted in anterograde strengthening of items encoded during extinction. Our follow-up control experiment showed that this anterograde memory effect was also time-dependent and thus required a (re)consolidation process. One possibility is that the anterograde strengthening of memory reflects a test effect see refs 54 and 55. However, again, the reminder did not explicitly test episodic memory, but did enhance memory and not merely protect against forgetting, and generalized to non-cued items. A second possibility is that the reminder cue enhanced attention during subsequent extinction learning but we did not observe better learning during extinction as expressed in SCR, and an enhanced attention explanation would predict better memory on the immediate memory test, which we did not observe. A third possibility is that items from extinction are enhanced due to reconsolidation of the items from acquisition triggered by the reminder cue. Reconsolidation has been postulated to serve an updating mechanism^19^, during which an old memory is updated with new information. It is conceivable that the reactivation of old items and activation of new items within the reconsolidation window resulted in an integration of episodic memory. In sum, we suggests that the retrograde effects of a reminder cue on the time-dependent strengthening of episodic memory for threatening events is most in line with a reconsolidation explanation, however the unexpected anterograde strengthening of items from extinction is more difficult to explain and we can not fully exclude a testing effect. A critical task for future studies is to delineate whether the reminder cue results in a reconsolidation induces change in neural memory representations, a testing effect induced change in retrieval mechanisms, or a change in attention mechanisms that affect new learning.

Our results also speak to the efficacy of a reminder to renew flexibility within an episodic memory network. Previous studies on reconsolidation of episodic-like hippocampal dependent memories with laboratory animals used re-exposure to context as a reminder^26, 28, 29, 31, 44, 56^. Human studies on episodic memory reconsolidation have used a basket, which contained the to-be-tested memoranda, as a reminder^19, 22, 23^, used cues to fully retrieve autobiographical memories^24^, used word stems as a reminder of memorized words^20^, or the first slide of a memorized slide show as a reminder^21^, and the context in which the reminder is presented may be critical to initiate reconsolidation of episodic memory^23^. Studies in the threat conditioning domain have generally used single trials of the same stimulus as presented during acquisition as a reminder as longer presentations or repeated exposure result in extinction not reconsolidation^57^. We aimed to reactivate memory for a threatening category by presenting the novel most prototypical exemplar of the threat-conditioned category. We did so because prototypical exemplars of a category provide the strongest source of generalization^58^ and we hypothesized that using such an item as a reminder might result in the most wide-spread reactivation of the associative memory network. The use of the most prototypical category exemplar as a reminder may have been critical to renew memory flexibility and result in the selective retroactive strengthening of episodic memory (see Supporting Information). Our observations confirm previous research indicating that a reminder does not need to be the exact replica of the original learning experience^59^ and indicate that a reminder can renew flexibility throughout an associative episodic memory network that, following a recent proposal on the characteristic of cognitive structures of memory^60^, may be more schematic than categorical (see Supporting Information for discussion). Collectively, this suggests that a reminder should be brief and critical to a memory network to initiate reconsolidation of episodic memories.

Generalization of threat responses is a hallmark of fear- and anxiety-related disorders. Our results have implications for the ability to modify generalized threat responses. We found retention, extinction, and recovery of generalized threat responses to novel events related to the reinforced category, extending previous findings that showed acquisition of generalized threat responses to category items^32, 33, 61^. Our results indicate that extinction, which is thought to underlie exposure treatment, is effective to reduce generalized threat responses and does not necessarily require re-exposure to the original threatening events. This finding is relevant because it is rarely possible to perform exposure treatment to the original events that induced trauma or to all events that evoke threat responses. However, akin to research using threat conditioning with simple stimuli we found that extinguished threat responses can be reinstated, limiting the clinical effectiveness of extinction training. Furthermore, in contrast to research using threat conditioning with simple stimuli^13, 34, 62–64^, we found that an isolated reminder of memory for a threatening category before extinction did not prevent the recovery of generalized threat responses (see also Supporting Information for our pilot study). This finding is in line with other research that found recovery of threat responses to simple stimuli following a reminder-extinction procedure in humans^41–43^. The use of a category threat conditioning may have increased the likelihood of threat recovery. As all items are novel and unique, participants have no experience whether a particular item is threatening or not and may therefore approach any new item of the threat category with caution. Our associative context task revealed that participants were well able to remember which category was associated with shocks but poor at identifying which particular items had been paired with a shock. This finding indicates that participants do not remember individual items-shock pairing but respond to items as part of their category membership. As participants at the time of the recovery test still remember which category was associated with a shock the activation of the category representation by a given item may evoke a threat response. Note, however, that this is true for both the reminder and no-reminder condition and can therefore not explain the absence of a difference in threat recovery between groups. Finally, we note that our item recognition, temporal- and associative context tests tested memory at a item level whereas our recovery test tested memory at a category level, possibly explaining differences between the observed effects of the isolated reminder on episodic memory and generalized threat responses. However, we also observe strengthening of episodic memory by the reminder on the shock estimation test, which also tests memory at a category level. For future studies it would be interesting to investigate if recovery would also be observed if participants were presented with the original items from acquisition. To conclude, the effectiveness of the reminder-extinction procedure to prevent the recovery of *generalized* threat responses may thus be limited.

A critical question for future research pertains to the optimal conditions that allow the renewal of memory flexibility and modification of threat responses. We speculate that our inability to attenuate generalized threat responses following a reminder may have resulted from a failure to renew flexibility of a sufficient number of item-US associations or of an association between the US and a higher-order representation of the category. Research that has used multiple conditioned CS+ s indicates that interventions only attenuate threat responses to a reminded CS+ but not a non-reminded CS+^13, 48^ or indirectly reminded CSs^65^. Interestingly, interventions following the use of the US as a reminder can attenuate threat responses to multiple CS+ s associated with the US^66^. It would be intriguing for future research to test if using a US as a reminder of memory for a threatening category would allow interventions to prevent the recovery of generalized threat responses. Further, we speculate that the increase in episodic memory demands may have resulted in a threat memory representation that is intrinsically less flexible. The formation and storage of threat memories for simple stimuli are supported by plasticity in a relative small number of neurons in the amygdala^67^. The formation of a category threat memory involves plasticity in a large region of representational neocortex^61^. Differences in neural architecture or molecular mechanisms between brain regions may explain limitations in the renewal of memory flexibility and the possibility to attenuate generalized threat responses after a reminder^3, 68^. It would be interesting to test if other interventions (e.g. medication or electrical stimulation) can attenuate generalized threat responses. Finally, the reminder may have initiated reconsolidation of generalized threat responses but not have strengthened responses, unlike our observations for episodic memory. Interestingly, strengthening of episodic memory due to reconsolidation – in the absence of an intervention method - has only been reported for hippocampal-dependent forms of memory^26, 28, 44^ not amygdala-dependent forms of memory. Furthermore, reconsolidation can catalyze systems-consolidation of episodic-like memories^44^ and as such specifically affect episodic memory for threatening events as tested here, which may depend on amygdala-hippocampal-cortical interactions^61, 69^. This strengthening of episodic memory via reconsolidation may also explain why threat responses are less sensitive to disruption when they involve greater episodic memory demands.

To conclude, by using more comprehensive analyses of episodic memory within a Pavlovian threat-conditioning paradigm we provide evidence that an isolated reminder can renew flexibility of episodic memory and result in selective strengthening of episodic memory for events associated with a Pavlovian learning experience. Our results unite findings on the flexibility of episodic memory following a reminder from studies on reconsolidation that have used threat-conditioning paradigms and those that specifically focussed on episodic memory, and bridge the gap between studies investigating reconsolidation of episodic memory in humans and studies investigating reconsolidation of threat responses in animals. We also demonstrated that a reminder before extinction did not prevent the recovery of generalized threat responses. This implies that the effectiveness of the reminder-intervention procedures to target and change specific learned responses and memories that contribute to maladaptive mental states and behaviours may be limited. Together our results provide supporting evidence for reconsolidation of episodic memories and indicate that reconsolidation may allow for the selective strengthening of relevant episodic memories, highlighting the flexible nature of memory.

## Methods

### Participants

Forty-eight healthy adult participants were included in the study (see Supporting Information for inclusion/exclusion criteria). The study was approved by the University Committee on Activities Involving Human Subjects at New York University. All participants provided written informed consent. All methods were carried out in accordance with the Declaration of Helsinki. In a between-subjects design participants were randomly assigned to the reminder or no-reminder group. To ensure that we could assess the effect of an isolated reminder on episodic memory for an aversive Pavlovian learning experience, participants were excluded from analyses if differential conditioning could not be established because electrodermal recordings failed during the tasks due to technical problems (N = 6) or if participants did not acquire threat responses (mean SCR to CS+ > mean CS− during late phase of conditioning, N = 4). The final reminder group comprised 18 participants (7 males, 11 females; age: 21.1667 ± 2.0934, range: 18–26) and the no-reminder group 20 participants (10 males, 10 females; age: 22.900 ± 2.5526, range: 19–29). For the follow-up control experiment an additional twenty-two participants were included, 3 did not complete the study, 3 did not acquire differential threat responses. The reminder immediate memory group thus comprised 16 participants (males 3, females 13; age: 20.6350 ± 2.8773, range: 18–28), but as one participant did acquire differential threat responses but SCR measurements saturated on day 2 this participant was taken along for episodic memory analyses but could not be included for SCR analyses. There were no differences in age, gender, or shock intensity setting between groups.

### Tasks

The study was conducted over three consecutive days. Day 1: acquisition. Day 2: An isolated reminder (reminder group) or not (no-reminder group) and extinction. Day 3: reinstatement, recovery test, and episodic memory tests. The experiment was conducted in the same test room on each day and SCR electrodes (see below) were attached during all conditioning tasks (acquisition, reminder, extinction, reinstatement, recovery test). In a follow-up control experiment a group of participants followed the exact same procedure as our reminder group, but here memory was tested immediately after the reminder-extinction procedure on day 2 (Fig. 1).

#### Acquisition

Day 1 consisted of a category discrimination delay-conditioning task with partial reinforcement^13, 32, 37^. Participants were instructed that they would see on a computer screen pictures of fish and pictures of birds while they could receive shocks, to pay attention to the computer screen, and told that a relationship existed between the stimuli and the shocks. The level of shock intensity was calibrated before the start of the experiment using an ascending staircase procedure. Participants were instructed that the intensity should be set at a level that was “maximally uncomfortable without being painful” and to stop the calibration once the intensity had reached this level. The staircase procedure started with a low voltage setting near a perceptual threshold and was increased after each shock until participants told the experimenter to stop. The shock intensity was kept at the same level for the remainder of the study. The CSs were trial-unique images of fish and birds presented on a grey background for 4 seconds, with an 11, 12, or 13 seconds inter-trial interval during which a fixation cross was presented. The US was a 200 ms mild electric shock delivered to the wrist (Grass Medical Instruments stimulator, West Warwick, Rhode Island) that co-terminated with the CS+. During acquisition there were 10 presentations of the CS+ that co-terminated with the US, and intermixed with an additional 10 CS+ trials (50% reinforcement rate) and 20 CS− trials that did not co-terminate with the US. The first trial was always a CS+ that co-terminated with the US and therefore excluded from SCR analyses accounting for orienting responses. For all tasks the order of the different trial types was pseudo-randomized so that no more than 2 trials of the same type occurred in a row.

#### Reminder and extinction

On day 2 only the reminder group was reminded of the CS+ category by the isolated presentation of a novel item that was the most prototypical exemplar of the CS+ category. Both groups then watched a video (BBC Planet Earth) for 10 min before extinction training on novel unique items. Extinction included 12 (reminder group) or 13 CS+ trials (no-reminder group) and 12 CS− trials without the US. As the reminder trial is also a non-reinforced CS+ trial, the addition CS+ trial during extinction was included for the no-reminder group to keep the total number of non-reinforced items equal as in previous studies^13, 34^. The first trial was always a CS− trial and disregarded to account for the orienting response^13, 37^.

#### Memory tests

The reminder and no-reminder group of the main experiment conducted memory tests on day 3, whereas the reminder group in the follow-up control experiment completed these on day 2. First, four unsignalled USs (ITIs 20, 30, 25, 15 seconds) were administered to reinstate threat responses. Next, participants underwent a recovery test on novel and unique items including 10 CS+ and 10 CS− trials. During all sessions (acquisition, reminder, extinction, and recovery) with the exception of the breaks, SCR and shock electrodes were attached to the participants and the shock stimulator was set to the ‘on’ position. Following the recovery test participants performed a surprise subsequent item recognition memory task. All unique items from acquisition (20 CS+, 20 CS−) and extinction (12 CS+, 12 CS−) were again presented intermixed with an equal number of novel items for each category. Participants indicated whether stimuli were old or new on a six-point likert scale (very sure old, sure old, probably old, probably new, sure new, very sure new). Next participants performed a temporal context memory test. All unique items from acquisition and extinction were again presented (without novel items), as during the item recognition test, and participants indicated whether stimuli had been presented on day 1 or on day 2 on a six-point likert scale (very sure day 1, sure day 1, probably day 1, probably day 2, sure day 2, very sure day 2). Participants then performed an associative context memory test. All unique items from acquisition and extinction were again presented, as during the temporal context memory test, and participants indicated whether the stimuli had been followed by a shock on a six-point likert scale (very sure shock, sure shock, probably shock, probably no shock, sure no shock, very sure no shock). For all memory tests images were presented according to two randomization orders for each category and two orders for CS type so that the randomization was balanced across groups and across CS categories. Images were presented for 3 seconds with a 1 second ISI. Finally participants completed the shock estimation memory questionnaire^14^, on which participants estimated the number of shock they thought had followed images of either category.

### Episodic memory analyses

D-prime and criterion scores were calculated^70^ for the item recognition memory task for CS+ and CS− items from acquisition and extinction separately. Hit rate was calculated for CS+ and CS− items separately as the total number of ‘very sure old’, ‘sure old’, ‘probably old’ responses divided by the total number of response to old CS+ or CS− items. False alarm rate (FA) was calculated as the total number of ‘very sure old’, ‘sure old’, ‘probably old’ responses divided by the total number of response to new CS+ or CS− items. If hit or false alarm rates were equal to one or zero the scores were replaced with 1–1/(2 * number of responses) and 1/(2 * number of responses), respectively, where responses refer to the total number of trials to which a participant made a button press. Hit and false alarm rates were z-transformed by taking the inverse of the normal cumulative distribution with mean zero and standard deviation one. D-prime was then calculated as zHit-zFA. Criterion was calculated as −0.5*(zHit + zFA).

On the context memory tests no new items were presented. We therefore calculated for the temporal context test a corrected memory score for CS+ and CS− items as the difference between z-transformed correct and incorrect responses, and considering difficulty of the task limited analyses to very sure and sure responses: for acquisition (very sure day 1+ sure day 1) − (sure day 2 + very sure day 2), and for extinction (very sure day 2+ sure day 2) − (sure day 1 + very sure day 1). For the associative context memory test we similarly calculated a corrected memory score for CS+ items that were followed by a shock during acquisition, and for CS+ items and CS− items from acquisition and extinction that had not been followed by a shock as the difference between z-transformed correct and incorrect responses; for the CS+ items from acquisition that had been followed by a shock (very sure shock + sure shock) − (sure no shock + very sure no shock), and for the other items (very sure no shock + sure no shock) − (sure shock + very sure day shock).

For the shock estimation memory test the number of estimated shocks for the CS+ and CS− category served as US estimation scores^14^.

### SCR acquisition and analyses

SCR was assessed with pre-gelled snap electrodes (BIOPAC EL509) placed on the hypothenar eminence of the palmar surface of the non-dominant hand. Data were collected using BIOPAC MP-100 System (Goleta, CA) and continuously recorded at 200 samples per second. SCR data was assessed using an in-house analysis program written in Matlab (the MathWorks) and using FieldTrip^71^. Data were low-pass filtered at 5 Hz. Responses were determined for each trial as the peak-to-peak amplitude difference in skin conductance of the largest deflection in the latency window from 0–8 s after stimulus onset, i.e. maximum SCR value minus the minimum value that preceded the maximum value in time. The raw skin conductance responses were square root transformed and analyses were restricted to non-reinforced trials only, in accordance with previous literature^14, 32, 72, 73^ where we note that previous reports have found no difference in responses to reinforced and non-reinforced items^32, 33, 61^. Mean scores for the early (first half of the trials of a task) and late (second half of the trials of a task) phase were calculated for each task. A delta-recovery score was calculated as the difference between SCR to the first trial of the recovery test and the last trial of extinction for the CS+ and CS− separately.

### Statistics

The d-prime, criterion, and corrected temporal- and associative context memory scores were subjected to task (acquisition, extinction) × CStype (CS+, CS−) × group (reminder, no-reminder) repeated measures ANOVAs. The shock estimation memory scores were subjected to a CStype (CS+, CS−) × group (reminder, no-reminder) repeated measures ANOVA. For analyses of SCR data from acquisition and extinction we ran phase (early, late) × CStype (CS+, CS−) × group (reminder, no-reminder) repeated measures ANOVAs (see Table S1). The delta recovery scores were entered into a CStype (CS+, CS−) × group (reminder, no-reminder) repeated measures ANOVA. Statistics were Greenhouse-Geisser or Huyn-Feldt corrected for non-sphericity when appropriate. For the follow-up control experiment we followed the same analyses comparing the original reminder group with the reminder immediate memory test group. Significant findings from ANOVAs were followed up by paired- and independent samples t-tests. As we were particularly interested in the retroactive effect of a reminder on episodic memory encoded 24 h hours earlier, we planned comparisons on the items from acquisition specifically. Otherwise tests are indicated as post-hoc t-tests. Relevant mean ± s.e.m. values are provided in figure legends.

### Significance statement

Contemporary research suggests that calling a memory to mind can temporarily renew its flexibility, referred to as reconsolidation, and make it vulnerable to alteration. Most research on reconsolidation has focussed on altering learned threat responses. But because of differences in study approaches it is unclear if other components of a memory, such as knowing the events that make up an episodic experience, can also become flexible. Here we united the different approaches and show that a reminder can renew flexibility of episodic memory for threatening events. Our results highlight the flexible nature of memory and suggest that targeted interventions may also alter episodic components of our memories.

## Electronic supplementary material

Supplementary Information

## References

[1] McGaugh JL (2000). Memory–a Century of Consolidation. Science.

[2] Nader K, Hardt O (2009). A single standard for memory: the case for reconsolidation. Nat Rev Neurosci.

[3] Alberini CM, LeDoux JE (2013). Memory reconsolidation. Current biology: CB.

[4] Schiller, D. & Phelps, E. A. Does reconsolidation occur in humans? *Front*. *Behav*. *Neurosci*. **5**, doi:10.3389/fnbeh.2011.00024 (2011). 10.3389/fnbeh.2011.00024 PMC3099269 21629821

[5] Kroes MCW, Fernández G (2012). Dynamic neural systems enable adaptive, flexible memories. Neurosci. Biobehav. Rev..

[6] Sara SJ, Hars B (2006). In memory of consolidation. Learn. Mem..

[7] Squire LR (1992). Memory and the hippocampus: A synthesis from findings with rats, monkeys, and humans. Psychol. Rev..

[8] Henke K (2010). A model for memory systems based on processing modes rather than consciousness. Nat. Rev. Neursci..

[9] Tulving, E. In *Organization of memory* (eds Tulving, E. & Donaldson, W.) 381–403 (Academic Press, 1972).

[10] Davachi L (2006). Item, context and relational episodic encoding in humans. Curr. Opin. Neurobiol..

[11] Dudai Y (2004). The Neurobiology of Consolidations, Or, How Stable is the Engram?. Annu. Rev. Psychol..

[12] Kindt M, Soeter M, Vervliet B (2009). Beyond extinction: erasing human fear responses and preventing the return of fear. Nat. Neurosci..

[13] Schiller D (2010). Preventing the return of fear in humans using reconsolidation update mechanisms. Nature.

[14] Kroes MCW (2015). How Administration of the Beta-Blocker Propranolol Prior to Extinction can Prevent the Return of Fear. Neuropsychopharmacology.

[15] Funayama ES, Grillon C, Davis M, Phelps EA (2001). A Double Dissociation in the Affective Modulation of Startle in Humans: Effects of Unilateral Temporal Lobectomy. J. Cogn. Neurosci..

[16] Ohman A, Soares JJF (1994). “Unconscious anxiety”: phobic responses to masked stimuli. J. Abnorm. Psychol..

[17] Raio CM, Carmel D, Carrasco M, Phelps EA (2012). Nonconscious fear is quickly acquired but swiftly forgotten. Curr. Biol..

[18] Schultz DH, Helmstetter FJ (2010). Classical conditioning of autonomic fear responses is independent of contingency awareness. J. Exp. Psychol. Anim. Behav. Process..

[19] Hupbach A, Gomez R, Hardt O, Nadel L (2007). Reconsolidation of episodic memories: A subtle reminder triggers integration of new information. Learn. Mem..

[20] Strange BA, Kroes MC, Fan J, Dolan RJ (2010). Emotion causes targeted forgetting of established memories. Front. Behav. Neurosci..

[21] Kroes MCW (2014). An electroconvulsive therapy procedure impairs reconsolidation of episodic memories in humans. Nat. Neurosci..

[22] Hupbach A, Gomez R, Nadel L (2009). Episodic memory reconsolidation: updating or source confusion?. Memory.

[23] Hupbach A, Hardt O, Gomez R, Nadel L (2008). The dynamics of memory: Context-dependent updating. Learn. Mem..

[24] Schwabe L, Wolf OT (2009). New episodic learning interferes with the reconsolidation of autobiographical memories. PLoS One.

[25] Kroes MCW, Strange BA, Dolan RJ (2010). β-Adrenergic Blockade during Memory Retrieval in Humans Evokes a Sustained Reduction of Declarative Emotional Memory Enhancement. J. Neurosci..

[26] Inda MC, Muravieva EV, Alberini CM (2011). Memory Retrieval and the Passage of Time: From Reconsolidation and Strengthening to Extinction. J. Neurosci..

[27] Debiec J, LeDoux JE, Nader K (2002). Cellular and Systems Reconsolidation in the Hippocampus. Neuron.

[28] Lee, J. L. C. Memory reconsolidation mediates the strengthening of memories by additional learning. *Nat*. *Neurosci*. **11**, 1264–1266, http://www.nature.com/neuro/journal/v11/n11/suppinfo/nn.2205_S1.html (2008). 10.1038/nn.2205 18849987

[29] Milekic MH, Alberini CM (2002). Temporally Graded Requirement for Protein Synthesis following Memory Reactivation. Neuron.

[30] Otis JM, Mueller D (2011). Inhibition of beta-adrenergic receptors induces a persistent deficit in retrieval of a cocaine-associated memory providing protection against reinstatement. Neuropsychopharmacology.

[31] Morris RG (2006). Memory reconsolidation: sensitivity of spatial memory to inhibition of protein synthesis in dorsal hippocampus during encoding and retrieval. Neuron.

[32] Dunsmoor JE, Martin A, LaBar KS (2012). Role of conceptual knowledge in learning and retention of conditioned fear. Biol. Psychol..

[33] Dunsmoor, J. E., Murty, V. P., Davachi, L. & Phelps, E. A. Emotional learning selectively and retroactively strengthens memories for related events. *Nature* (2015). 10.1038/nature14106 PMC4432479 25607357

[34] Monfils M-H, Cowansage KK, Klann E, LeDoux JE (2009). Extinction-Reconsolidation Boundaries: Key to Persistent Attenuation of Fear Memories. Science.

[35] Tedesco V, Roquet RF, DeMis J, Chiamulera C, Monfils MH (2014). Extinction, applied after retrieval of auditory fear memory, selectively increases zinc-finger protein 268 and phosphorylated ribosomal protein S6 expression in prefrontal cortex and lateral amygdala. Neurobiol. Learn. Mem..

[36] Shumake, J. & Monfils, M. H. Assessing fear following retrieval + extinction through suppression of baseline reward seeking vs. freezing. *Front*. *Behav*. *Neurosci*. **9**, doi:10.3389/fnbeh.2015.00355 (2015). 10.3389/fnbeh.2015.00355 PMC4688362 26778985

[37] Schiller D, Kanen JW, LeDoux JE, Monfils M-H, Phelps EA (2013). Extinction during reconsolidation of threat memory diminishes prefrontal cortex involvement. Proc. Natl. Acad. Sci. USA.

[38] Clem RL, Huganir RL (2010). Calcium-Permeable AMPA Receptor Dynamics Mediate Fear Memory Erasure. Science.

[39] Xue Y-X (2012). A Memory Retrieval-Extinction Procedure to Prevent Drug Craving and Relapse. Science.

[40] Lee, H. J., Haberman, R. P., Roquet, R. & Monfils, M. H. Extinction and retrieval + extinction of conditioned fear differentially activate medial prefrontal cortex and amygdala in rats. *Front*. *Behav*. *Neurosci*. **9**, doi:10.3389/fnbeh.2015.00369 (2016). 10.3389/fnbeh.2015.00369 PMC4722140 26834596

[41] Kindt M, Soeter M (2013). Reconsolidation in a human fear conditioning study: A test of extinction as updating mechanism. Biol. Psychol..

[42] Golkar, A., Bellander, M., Olsson, A. & Öhman, A. Are fear memories erasable? –reconsolidation of learned fear with fear relevant and fear-irrelevant stimuli. *Front*. *Behav*. *Neurosci*. **6**, doi:10.3389/fnbeh.2012.00080 (2012). 10.3389/fnbeh.2012.00080 PMC3501228 23181015

[43] Klucken T (2016). No evidence for blocking the return of fear by disrupting reconsolidation prior to extinction learning. Cortex.

[44] Ye, X., Kapeller-Libermann, D., Travaglia, A., Inda, M. C. & Alberini, C. M. Direct dorsal hippocampal-prelimbic cortex connections strengthen fear memories. *Nat*. *Neurosci*. **20**, 52–61, doi:10.1038/nn.4443, http://www.nature.com/neuro/journal/v20/n1/abs/nn.4443.html-supplementary-information (2017). 10.1038/nn.4443 PMC5191950 27869801

[45] Finsterwald C, Steinmetz AB, Travaglia A, Alberini CM (2015). From Memory Impairment to Posttraumatic Stress Disorder-Like Phenotypes: The Critical Role of an Unpredictable Second Traumatic Experience. J. Neurosci..

[46] de Voogd, L. D., Fernández, G. & Hermans, E. J. Disentangling the roles of arousal and amygdala activation in emotional declarative memory. *Soc*. *Cogn*. *Affect*. *Neurosci*., doi:10.1093/scan/nsw055 (2016). 10.1093/scan/nsw055 PMC5015804 27217115

[47] Morris RGM, Frey U (1997). Hippocampal synaptic plasticity: role in spatial learning or the automatic recording of attended experience?. Philosophical Transactions of the Royal Society B: Biological Sciences.

[48] Nader K, Schafe GE, LeDoux JE (2000). Fear memories require protein synthesis in the amygdala for reconsolidation after retrieval. Nature.

[49] Nader K, Einarsson EÖ (2010). Memory reconsolidation: an update. Ann. N. Y. Acad. Sci..

[50] Kroes, M. C. W., Schiller, D., LeDoux, J. E. & Phelps, E. A. In *Translational Neuropsychopharmacology* (eds Trevor, W. Robbins & Barbara, J. Sahakian) 197–230 (Springer International Publishing, 2016).

[51] van den Broek G (2016). Neurocognitive mechanisms of the “testing effect”: A review. Trends in Neuroscience and Education.

[52] Roediger HL, Karpicke Jeffrey D (2006). Test-Enhanced Learning. Psychological Science.

[53] Chan JCK, McDermott KB, Roediger Iii HL (2006). Retrieval-induced facilitation: Initially nontested material can benefit from prior testing of related material. J. Exp. Psychol. Gen..

[54] Cho KW, Neely JH, Crocco S, Vitrano D (2017). Testing enhances both encoding and retrieval for both tested and untested items. The Quarterly Journal of Experimental Psychology.

[55] Bacso SA, Marmurek HHC (2016). Testing effects of free recall on organization in whole/part and part/whole transfer. Acta Psychol. (Amst)..

[56] Otis JM, Mueller D (2011). Inhibition of β-Adrenergic Receptors Induces a Persistent Deficit in Retrieval of a Cocaine-Associated Memory Providing Protection against Reinstatement. Neuropsychopharmacology.

[57] Eisenberg M, Kobilo T, Berman DE, Dudai Y (2003). Stability of Retrieved Memory: Inverse Correlation with Trace Dominance. Science.

[58] Dunsmoor JE, Murphy GL (2014). Stimulus Typicality Determines How Broadly Fear Is Generalized. Psychological Science.

[59] Soeter, M. & Kindt, M. Retrieval cues that trigger reconsolidation of associative fear memory are not necessarily an exact replica of the original learning experience. *Front*. *Behav*. *Neurosci*. **9**, doi:10.3389/fnbeh.2015.00122 (2015). 10.3389/fnbeh.2015.00122 PMC4435076 26042008

[60] Ghosh VE, Gilboa A (2014). What is a memory schema? A historical perspective on current neuroscience literature. Neuropsychologia.

[61] Dunsmoor JE, Kragel PA, Martin A, LaBar KS (2014). Aversive Learning Modulates Cortical Representations of Object Categories. Cereb. Cortex.

[62] Agren T (2012). Disruption of Reconsolidation Erases a Fear Memory Trace in the Human Amygdala. Science.

[63] Agren, T., Furmark, T., Eriksson, E. & Fredrikson, M. Human fear reconsolidation and allelic differences in serotonergic and dopaminergic genes. *Transl Psychiatry* **2**, e76, http://www.nature.com/tp/journal/v2/n2/suppinfo/tp20125s1.html (2012). 10.1038/tp.2012.5 PMC3309551 22832813

[64] Steinfurth EC (2014). Young and old Pavlovian fear memories can be modified with extinction training during reconsolidation in humans. Learn. Mem..

[65] Debiec J, Doyere V, Nader K, LeDoux JE (2006). Directly reactivated, but not indirectly reactivated, memories undergo reconsolidation in the amygdala. Proc. Natl. Acad. Sci. USA.

[66] Liu J (2014). An Unconditioned Stimulus Retrieval Extinction Procedure to Prevent the Return of Fear Memory. Biol. Psychiatry.

[67] Fanselow MS, LeDoux JE (1999). Why We Think Plasticity Underlying Pavlovian Fear Conditioning Occurs in the Basolateral Amygdala. Neuron.

[68] Kroes, M. C. W., Schiller, D., LeDoux, J. E. & Phelps, E. A. 1–34 (Springer International Publishing).

[69] de Voogd LD, Fernández G, Hermans EJ (2016). Awake reactivation of emotional memory traces through hippocampal–neocortical interactions. Neuroimage.

[70] Yonelinas AP, Parks CM (2007). Receiver operating characteristics (ROCs) in recognition memory: a review. Psychol. Bull..

[71] Oostenveld, R., Fries, P., Maris, E. & Schoffelen, J.-M. Fieldtrip: Open Source Software for Advanced Analysis of MEG, EEG, and Invasive Electrophysiological Data. *Computational Intelligence and Neuroscience* Article ID 156869, 1–9, doi:10.1155/2011/156869 (2011). 10.1155/2011/156869 PMC3021840 21253357

[72] Milad MR (2007). Recall of Fear Extinction in Humans Activates the Ventromedial Prefrontal Cortex and Hippocampus in Concert. Biol. Psychiatry.

[73] Schiller D, Levy I, Niv Y, LeDoux JE, Phelps EA (2008). From Fear to Safety and Back: Reversal of Fear in the Human Brain. J. Neurosci..

