# Supplementary material for: A reminder before extinction strengthens episodic memory via reconsolidation but fails to disrupt generalized threat responses
Source: Sci Rep. 2017 Sep 7;7:10858. doi: 10.1038/s41598-017-10682-7 (PMC5589753; doi:10.1038/s41598-017-10682-7)
Supplement: Supplementary file 1 — Supplementary Information [file 41598_2017_10682_MOESM1_ESM.docx]

Supporting Information

**A reminder before extinction strengthens episodic memory via reconsolidation but fails to disrupt generalized threat responses.**

Marijn CW Kroes^1,2,*^, Joseph E Dunsmoor^1^, Qi Lin^1^, Michael Evans^1^, and Elizabeth A Phelps^1,2,3*^

^1^Department of Psychology, New York University, New York, NY 10003, United States; ^2^Center for Neural Science, New York University, New York, NY 10003, United States; ^3^Nathan Kline Institute, Orangeburg, NY 10962, United States

#

# Supporting methods main experiment

## Participants

Participants were free of neurological, cardiovascular, endocrine or psychiatric history, were right-hand dominant, had normal or corrected-to-normal vision and hearing, and had no history with psychotropic medication, did not use recreational drugs weekly or more, had not used over-the-counter medication in the 72 hours, or alcohol in the 24 hours prior to study, with the exception of oral contraceptives and paracetamol.

## Design

The main experiment was conducted over three consecutive days: day 1, acquisition; day 2, isolated reminder (reminder group) or no reminder (no-reminder group) and extinction; and day 3, memory tests.

# Supporting results main experiment

#### Supporting Table 1: Results of ANOVA tests on SCR data per task

|  | Acquisition | Extinction | ΔReinstatement |
| --- | --- | --- | --- |
| Phase | n.s. | F_1, 36_=32.943, p<0.001  η^2^=0.478 | n.s. |
| CStype | F_1, 36_=73.281, p<0.001, η^2^=0.671 | F_1, 36_=33.872, p<0.001, η^2^=0.485 | F_1, 36_=11.377, p=0.002, η^2^=0.240 |
| Group | n.s. | n.s. | n.s. |
| Phase x CStype | F_1, 36_=23.431, p<0.001, η^2^=0.394 | F_1, 36_=3.356, p=0.075, η^2^=0.085 | n.s. |
| Phase x Group | n.s. | n.s. | n.s. |
| CStype x Group | n.s. | n.s. | n.s. |
| Phase x CStype x Group | n.s. | n.s. | n.s. |

Results of repeated measures ANOVAs on SCRs. For acquisition and extinction SCRs were average over the first half (early phase) and second half (late phase) of the task and we ran a phase (early, late) x CStype (CS+, CS-) x group (reminder, no-reminder) repeated measures ANOVA. Acquisition early phase (CS+ trial 1-5; CS- trial 1-10), late phase (CS+ trial 6-10; CS- trial 11-20) of task). Extinction early phase (CS+ trial 1-6; CS- trial 2-6), late phase (CS+ trial 7-12; CS- trial 7-12 of task). The delta recovery index reflected the difference in responses to the last trial of extinction and the first trial of the recovery test. The delta recovery scores were subjected to a CStype (CS+, CS-) x group (reminder, no-reminder) repeated measures ANOVA. n.s. = non-significant p>0.1.

#### Results associative context memory test.

We measured participant’s memory for the associative context of the items, i.e. whether an item had co-terminated with a shock or not, by calculating a corrected memory score (correct - incorrect responses) for CS+ and CS- items from acquisition and extinction separately (Figure S2). The corrected memory scores were submitted to a group (reminder, no-reminder) x task (acquisition, extinction) x CStype (CS+, CS-) repeated measures ANOVA. Memory performance for associative context marginally differed between groups (group x CStype: F_1, 36_=4.038, p=0.052, η^2^=0.101). The reminder group tended to have better memory for associative context across CS+US and CS+ items from acquisition and extinction. Across both groups participants accurately indicated that CS- items had not co-terminated with a shock. Yet participants were likely to indicate for any CS+ item that it had co-terminated with a shock, regardless whether the item had in fact co-terminated with a shock (CS+US) or not (CS+) during acquisition, or had been presented during extinction (task x CStype: F_1, 36_=10.169, p=0.003, η^2^=0.220; task: F_1, 36_=13.861, p=0.001, η^2^=0.278; CStype: F_1, 36_=673.294, p<0.001, η^2^=0.949). Participants in the reminder group, thus, tended to be better at identifying the CS+ items that had in fact co-terminated with shock, although both groups were inclined to respond that CS+ items had been followed by a shock. A group (reminder, reminder immediate memory test) x task (acquisition, extinction) x CStype (CS+, CS-) repeated measures ANOVA revealed no effects of group (task x CStype x group: F_1, 32_=2.278, p=0.141, η^2^=0.066). Thus we cannot establish that this effect was time-dependent.

#### Figure S1: Results associative context test

#
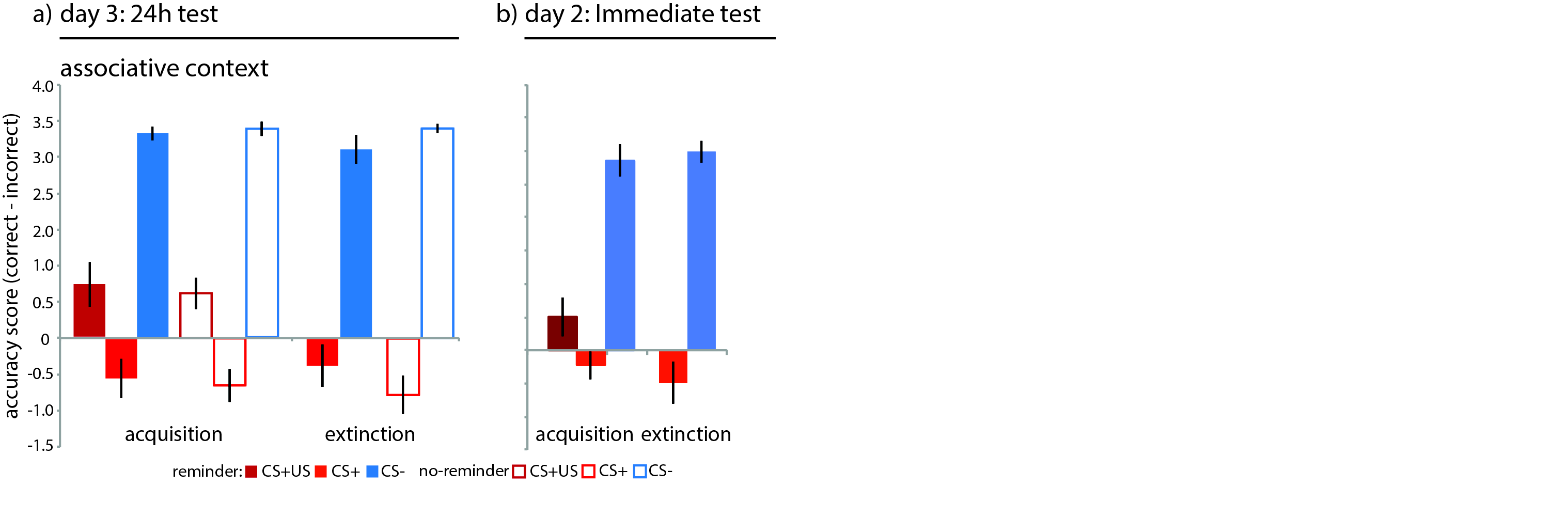


# Associative context memory results. A reminder resulted in marginally better memory for the associative context of items from the CS+ category. Follow-up one-sample t-test revealed that participants correctly indicated CS+US items to have been paired with shock (t(37)=3.653, p=0.001), but incorrectly indicated CS+ items from acquisition (t(37)=-3.433, p=0.001) and extinction (t(37)=-3.012, p=0.005) to have been paired with shock, and correctly indicated CS- items from acquisition (t(37)=40.817, p<0.001) and extinction (t(37)=26.607, p<0.001) not to have been paired with shock. Follow-up independent samples T-test on the average score across CS+US and CS+ items from acquisition and CS+ items from extinction indicated better memory performance in the reminder group at trend (t(36) =-1.848, p=0.073; no reminder: -0.0902 ± 0.0967; reminder: 0.1917 ± 0.1196), but no differences for the CS- items (t(36)=1.051, p=0.300; no-reminder: 3.1867 ± 0.0902; reminder: 2.9975 ± 0.1613). B) The immediate test group was not able to correctly indicate the CS+US items were paired with shock (t(15)=0.806, p=0.433), and did not incorrectly indicate CS+ items from acquisition (t(15)=-0.656, p=0.521) or extinction (t(15)=-0.762, p=0.458) to be paired with shock but was able to indicate that CS- items from acquisition (t(15)=13.781, p<0.001) and extinction (t(15)=18.089, p<0.001) were not paired with shock.

# Supporting methods pilot experiment

## Participants

Sixty healthy young participants with normal- or corrected-to-normal vision, normal uncorrected hearing, were included in the pilot study. The study was approved by the University Committee on Activities Involving Human Subjects at New York University. All participants provided written informed consent. Inclusion and exclusion criteria, randomization procedures were identical to that of the main experiment (see main text).

## Tasks

Identical to the main experiment, the pilot experiment was conducted over three consecutive days: day 1, acquisition; day 2, isolated reminder (reminder group) or no reminder (no-reminder group) and extinction; and day 3, memory tests (Figure S2). The pilot experiment did not include tests for temporal- and associative context memory.

#### Figure S2: Design pilot experiment


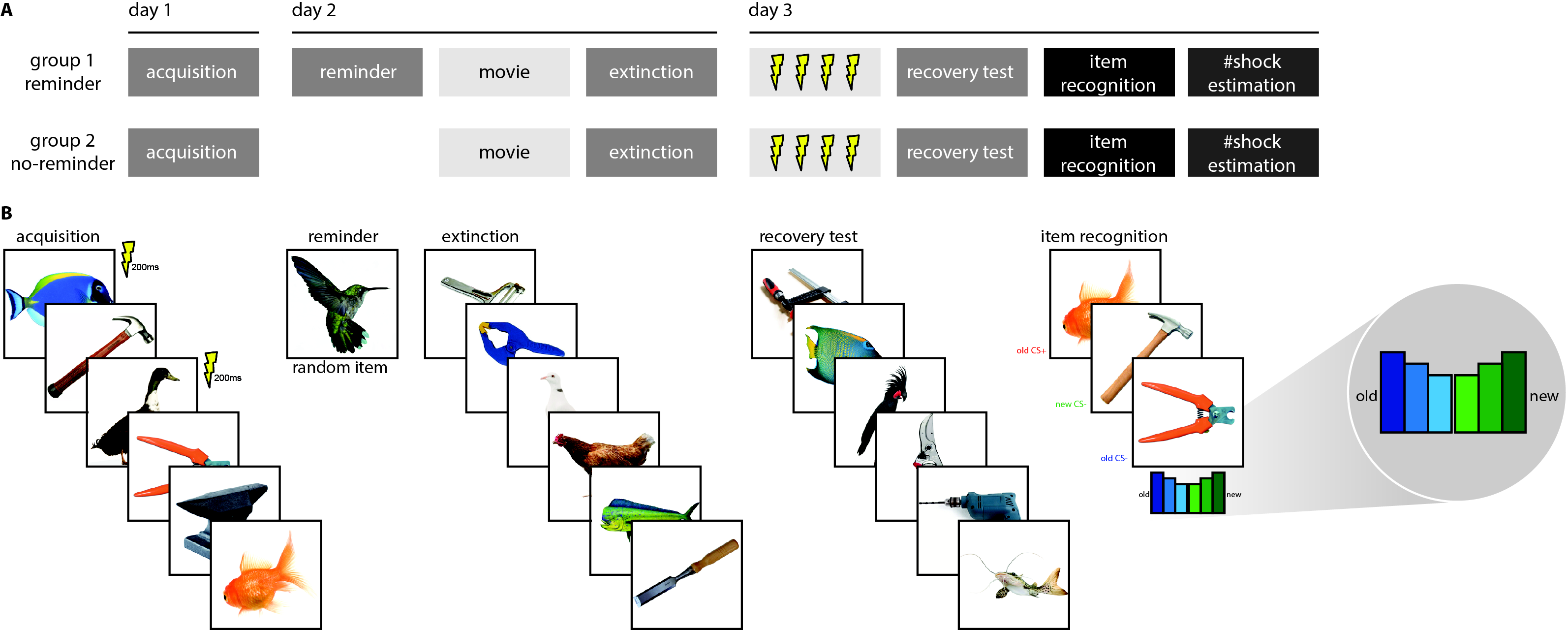


(A) Time line of pilot experiment and (B) task depiction. Over 3 consecutive days participants were differentially conditioned to trial-unique items from a specific category (animals versus tools, counter-balanced across participants) signalling threat (CS+) of transcutaneous electrical shock (US) and unique items signalling safety (CS−) on day 1. On day 2, one group received an isolated reminder (reminder group) but not the other (no-reminder group) by presentation of a trial-unique item randomly selected from the CS+ category. Next, both groups underwent extinction to novel trial-unique items. On day 3, following reinstatement, the recovery of generalized threat-related responses (skin conductance responses, SCR) was tested to trial-unique items. Finally, participants’ recognition memory for the items and the explicit memory for the number of received shocks were assessed. Responses during the recognition memory test were made on a six point likert scale (inset). Images were obtained from Lifeonwhite.com. This figure is not covered by the CC BY license. © lifeonwhite.com. All right reserved, used with permission.

### Day 1: Acquisition

Conditioning procedures were identical to that described for the main experiment with the following exceptions: Day 1 consisted of a category-conditioning task with partial reinforcement (38%). The CSs were unique images of objects and tools and participants were instructed that they would see on a computer screen pictures of animals and tools while they could receive shocks (Figure S2). During acquisition there were 10 presentations of each CS that did not co-terminate with the US, intermixed with an additional 6 CS+ trials that co-terminated with the US. The first trial was always a CS+.

### Day 2: Reminder and extinction

On day 2 only the reminder group was reminded of the CS+ category by presentation of a novel random item from the CS+ category for 4 seconds. Extinction included 10 (reminder group) or 11 CS+ trials (no-reminder group) and 11 CS− trials without the US.

### Day 3: Recovery and item memory test

On day 3 the recovery test included 10 novel and unique CS+ and 11 CS− items. The first trial was always a CS- trial and disregarded to account for the orienting response. Following the recovery test participants performed a surprise subsequent recognition memory task. All unique CS+ from acquisition (16) and CS- (10) items were again presented intermixed with an equal number of novel items for each category.

# Supporting results pilot experiment

## Results item recognition memory

We found no effect of conditioning or the reminder on item recognition memory (CStype: F_1, 34_=1.910, p=0.176, η^2^=0.053; group: F_1, 34_=1.819, p=0.186, η^2^=0.051; CStype x group: F_1, 34_=0.562, p=0.459, η^2^=0.016). A one-sample t-test on the mean d-prime score across all conditions revealed that participants were able to correctly discriminate old from new items (*t*(35)=13.561, p<0.001; 1.1612 ± 0.086).

## Results shock estimation memory

Across groups participants correctly indicated that shocks had occurred on day 1 during acquisition and had followed exemplars of the CS+ category but we found no effect of the reminder (day (F_2, 68_=90.189, p<0.001, η^2^=0.726; CStype: F_1, 34_=100.202, p<0.001, η^2^=0.747; day x CStype: F_2, 68_=69.897, p<0.001, η^2^=0.673). Paired samples t-test revealed that participants estimated having received more shocks to items of the CS+ category than CS- category on day 1 (*t*(35)=9.581, p<0.001; CS+: 7.1389, ± 0.6389; CS-: 0.4722 ± 0.1618).

## Results skin conductance responses

Both groups acquired differential conditioned threat-related responses on day 1 (Figure S3). A group (reminder, no-reminder) x phase (early phase, late phase of task) x CStype (CS+, CS-) repeated measures ANOVA on the mean SCRs revealed a main effect of phase (F_1, 35_=28.111, p<0.001, η^2^=0.445), CStype (F_1, 35_=22.843, p<0.001, η^2^=0.395), phase x CStype (F_1, 35_=23.098, p<0.001, η^2^=0.398), and group x phase x CStype interaction (F_1, 35_=7.230, p=0.011, η^2^=0.171). Follow-up independent t-test on the difference scores (CS+ - CS-) revealed no detectable differences in conditioned responses between groups in the early (*t*(35)=-1.557, p=0.129) or late phase (*t*(35)=0.681, p=0.501) of acquisition. Follow-up paired samples t-tests revealed greater difference scores during the late phase of acquisition than during the early phase (*t*(36)=-4.140, p<0.001; early phase: 0.1541 ± 0.0506; late phase: 0.3406 ± 0.0504), and greater responses to CS+ than CS- trial during the early phase (*t*(36)=3.045, p=0.004; CS+: 0.8072 ± 0.0676; CS-: 0.6530 ± 0.0539) and late phase (*t*(36)=6.764, p<0.001; CS+: 0.7127 ± 0.0762; CS-: 0.3720 ± 0.0556). Thus both groups acquired comparable generalized threat responses despite a difference in acquisition rate.

On day 2, we observed SCRs to the reminder trial in the reminder group. Both groups showed extinction of threat responses (Figure S3). A group (reminder, no-reminder) x phase (early phase, late phase of task) x CStype (CS+, CS-) repeated measures ANOVA on the mean SCRs revealed a main effect of phase (F_1, 35_=44.184, p<0.001, η^2^=0.558), CStype (F_1, 35_=27.837, p<0.001, η^2^=0.443), phase x CStype (F_1, 35_=4.651, p=0.038, η^2^=0.117), and group x phase interaction (F_1, 35_=4.437, p=0.042, η^2^=0.113). Follow-up independent samples T-tests revealed no detectable differences between groups for average responses to the CS+ and CS- combined in the early (*t*(35)=0.406, p=0.687) or late phase (*t*(35)=-1.778, p=0.084). Follow-up paired samples t-tests revealed greater difference scores during the early phase of extinction than during the late phase (*t*(36)=2.068, p=0.046; early phase: 0.3600 ± 0.0677; late phase: 0.2202 ± 0.0600), and greater responses to CS+ than CS- trial during the early phase (*t*(36)=5.316, p<0.001; CS+: 0.8174 ± 0.0794; CS-: 0.4573 ± 0.0510) and late phase (*t*(36)=3.670, p=0.001; CS+: 0.4529 ± 0.0630; CS-: 0.2327 ± 0.0375). To control for complete extinction we compared the last CS+ and CS- trials. A group x CStype repeated measure ANOVA revealed no main effect of group (F_1, 35_=1.630, p=0.210, η^2^=0.045), CStype (F_1, 35_=0.514, p=0.478, η^2^=0.014), or interaction (F_1, 35_=0.079, p=0.780, η^2^=0.002). Thus, on day 2 both groups showed comparable retention and extinction generalized threat responses.

On day 3 we observed recovery of generalized threat responses in both groups. A CStype (CS+, CS-) x group (reminder, no-reminder) repeated measures ANOVA on delta recovery scores revealed a main effect of CStype (F_1, 35_=7.489, p=0.010, η^2^=0.176), and no other main effect or interaction. A follow-up paired t-test revealed greater delta recovery for the CS+ than CS- (*t*(36)=2.798, p=0.008; CS+: 0.5762 ± 0.0854; CS-: 0.3169 ± 0.0680). Thus both groups showed comparable recovery of generalized threat-related responses.

#### Figure S3: Results skin conductance responses in pilot experiment


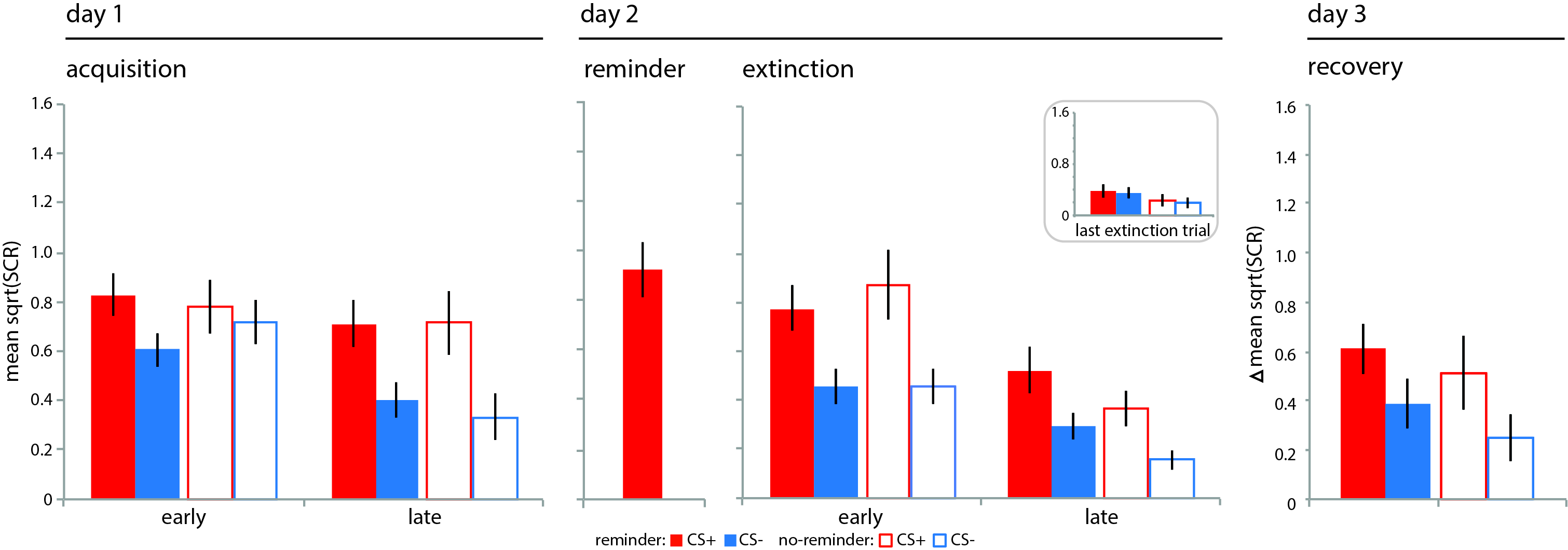


In the pilot experiment the aversive Pavlovian category threat-conditioning procedure resulted in the acquisition, retention and extinction of threat-related skin conductance responses. But an isolated reminder before extinction did not prevent the recovery of generalized threat responses one day later. Reminder group (solid bars, N=21), no-reminder group (open bars, N=16), CS+ (red), CS- (blue), error bars reflect SEM (see Supporting text for statistics).

# Supporting Discussion

In contrast to the main experiment we found no evidence that an isolated reminder affected episodic memory in the pilot experiment. We conceive at least three critical differences between the main and the pilot experiment that might explain this discrepancy. First, the pilot experiment followed trial numbers and reinforcement rate of previous reminder-extinction paradigms ^1,2^. Therefore we increased the number of trials and reinforcement rate in the main experiment. Second, in the pilot experiment memories were formed for broader categories (animals versus tools) compared to previous studies ^3-5^, therefore we narrowed the categories in the main experiment (birds versus fish). Third, in the pilot experiment the reminder trial was a randomly picked trial-unique exemplar of the CS+ category, whereas in the main experiment we used the most prototypical exemplar of the CS+ category as threat responses are known to more readily generalize to prototypical category exemplars ^6^. As the pilot experiment included less trials and item recognition memory was only tested for trials from acquisition, the item recognition memory test might have lacked adequate power to detect effects of conditioning and the reminder. The use of more trials, narrower categories, and a higher reinforcement rate, moreover, might have ensured that participants formed a better-defined category memory. In support of this idea, the SCR difference scores (CS+ - CS-) in the late phase of acquisition was greater in the main experiment than in the pilot experiment (*t*(73) = -3.405, p=0.001; pilot experiment: 0.1703 ± 0.0252; main experiment: 0.3211 ± 0.0362). A better-defined category memory in combination with the use of the most prototypical exemplar of the category as the reminder trial might have resulted in more optimal memory reactivation and a greater activation spread throughout the associative memory network. The results of our main experiment did replicate the finding from our pilot experiment that an isolated reminder of memory for an aversive category before extinction did not prevent the recovery of generalized threat-related skin conductance responses (For discussion see main text).

Recent research has shown that category threat-conditioning can result in a selective retroactive strengthening of episodic memory for related events dependent on a consolidation period ^5^. The results from our reminder group mirror these findings, but our control group did not show better memory for items of the reinforced category. A critical procedural difference is that here we tested episodic memory 24 hours after extinction training. During extinction participants learn that the contingency between the category and shock has decreased. Classically extinction is thought to result in interference learning ^7^. We speculate that extinction may have reduced the affective value associated with the category or resulted in interference between the associative value acquired during acquisition and extinction and changed the consolidation trajectory of episodic memory for exemplars related to the reinforced category. Furthermore, recall of a reminded memory during the reconsolidation window can prevent impairment of episodic memory ^8^. We conceive the possibility that the reminder procedure may have reactivated the affective relevance of the aversive category and protected episodic memory against extinction-induced changes. It remains to be determined if this explains why the no-reminder group did not show an episodic memory enhancement as a result of category threat-conditioning whereas the reminder group did. Note that this is why we speak of memory strengthening, as it is an open question whether the reminder protected episodic memory or resulted in enhancement or improvement.

The results of our episodic memory tests also speak to the cognitive structure of the memory representation. We used a category threat-conditioning procedure where the shared features between exemplars of the category form an associative network structure, and the importance of the category structure becomes apparent over multiple episodes (i.e. unique item presentations co-terminate with shock). Such memories are sensitive to the hierarchical structure of the category as threat responses more readily generalize from the most prototypical exemplars of a category ^6^. Beyond the cognitive structures of categories, the memory includes an association with the contingency of an aversive outcome that has a chronological organization (i.e. shocks follow the onset of category exemplars) that is adaptable as it is sensitive to extinction. The memory lacks unit detail as evidenced by the finding that participants were inclined to indicate that any item related to the reinforced category had been presented during conditioning and had co-terminated with a shock, regardless of whether this was true. Participants were, however, highly accurate in indicating that shocks had only co-terminated with exemplars of the reinforced category during acquisition on day 1. Following a recent proposal on the characteristics of cognitive structures of memory ^9^, the threat memory in the current study may thus be more schematic than categorical.

# Supporting References

1 Schiller, D., Kanen, J. W., LeDoux, J. E., Monfils, M.-H. & Phelps, E. A. Extinction during reconsolidation of threat memory diminishes prefrontal cortex involvement. *Proc. Natl. Acad. Sci. U. S. A.* **110**, 20040-20045, doi:10.1073/pnas.1320322110 (2013).

2 Schiller, D. *et al.* Preventing the return of fear in humans using reconsolidation update mechanisms. *Nature* **463**, 49-53 (2010).

3 Dunsmoor, J. E., Kragel, P. A., Martin, A. & LaBar, K. S. Aversive Learning Modulates Cortical Representations of Object Categories. *Cereb. Cortex* **24**, 2859-2872, doi:10.1093/cercor/bht138 (2014).

4 Dunsmoor, J. E., Martin, A. & LaBar, K. S. Role of conceptual knowledge in learning and retention of conditioned fear. *Biol. Psychol.* **89**, 300-305, doi:<http://dx.doi.org/10.1016/j.biopsycho.2011.11.002> (2012).

5 Dunsmoor, J. E., Murty, V. P., Davachi, L. & Phelps, E. A. Emotional learning selectively and retroactively strengthens memories for related events. *Nature* (2015).

6 Dunsmoor, J. E. & Murphy, G. L. Stimulus Typicality Determines How Broadly Fear Is Generalized. *Psychological Science* **25**, 1816-1821, doi:10.1177/0956797614535401 (2014).

7 Bouton, M. E. Context, time, and memory retrieval in the interference paradigms of Pavlovian learning. *Psychol. Bull.* **114**, 80-99 (1993).

8 Strange, B. A., Kroes, M. C., Fan, J. & Dolan, R. J. Emotion causes targeted forgetting of established memories. *Front. Behav. Neurosci.* **4**, 175, doi:10.3389/fnbeh.2010.00175 (2010).

9 Ghosh, V. E. & Gilboa, A. What is a memory schema? A historical perspective on current neuroscience literature. *Neuropsychologia* **53**, 104-114, doi:<http://dx.doi.org/10.1016/j.neuropsychologia.2013.11.010> (2014).
